# Hawkmoth Pheromone Transduction Involves G-Protein–Dependent Phospholipase Cβ Signaling

**DOI:** 10.1523/ENEURO.0376-24.2024

**Published:** 2025-03-05

**Authors:** Anna C. Schneider, Katrin Schröder, Yajun Chang, Andreas Nolte, Petra Gawalek, Monika Stengl

**Affiliations:** University of Kassel, Kassel 34132, Germany

**Keywords:** circadian clock, insect, olfaction

## Abstract

Evolutionary pressures adapted insect chemosensation to their respective physiological needs and tasks in their ecological niches. Solitary nocturnal moths rely on their acute olfactory sense to find mates at night. Pheromones are detected with maximized sensitivity and high temporal resolution through mechanisms that are mostly unknown. While the inverse topology of insect olfactory receptors and heteromerization with the olfactory receptor coreceptor suggest ionotropic transduction via odorant-gated receptor–ion channel complexes, contradictory data propose amplifying G-protein–coupled transduction. Here, we used in vivo tip-recordings of pheromone-sensitive sensilla of male *Manduca sexta* hawkmoths at specific times of day (rest vs activity). Since the olfactory receptor neurons distinguish signal parameters in three consecutive temporal windows of their pheromone response (phasic; tonic; late, long-lasting), respective response parameters were analyzed separately. Disruption of G-protein–coupled transduction and block of phospholipase C decreased and slowed the phasic response component during the activity phase of hawkmoths without affecting any other component of the response during activity and rest. A more targeted disruption of G_α_ subunits by blocking G_αo_ or sustained activation of G_αs_ using bacterial toxins affected the phasic pheromone response, while toxins targeting G_αq_ and G_α12/13_ were ineffective. Consistent with these data, the expression of phospholipase Cβ4 depended on zeitgeber time, which indicates circadian clock-modulated metabotropic pheromone transduction cascades that maximize sensitivity and temporal resolution of pheromone transduction during the hawkmoth's activity phase. Thus, discrepancies in the literature on insect olfaction may be resolved by considering circadian timing and the distinct odor response components.

## Significance Statement

Insect chemosensory transduction is typically thought to be ionotropic, but data from different insect species suggest that metabotropic olfactory signaling may occur, either alongside or instead of ionotropic mechanisms. Nocturnal moths, known for their extraordinarily sensitive pheromone-detecting olfactory receptor neurons, likely use metabotropic signal amplification. To overcome limitations of previous in vitro studies, we conducted tip-recordings of pheromone-sensitive sensilla in healthy hawkmoths at specific zeitgeber times (ZT). Disrupting G-protein and phospholipase Cβ signaling reduced sensitivity and altered response kinetics, revealing strict temporal control of transduction. Thus, contradictory findings in insect olfaction may be reconciled by considering diverse evolutionary pressures for distinct chemosensory signals in different species, ZT, and disparate odor response parameters.

## Introduction

Both male and female nocturnal *Manduca sexta* hawkmoths express daily rhythms in their mating behavior, which are synchronized via species-specific pheromones that are released exclusively at night. Superimposed on this slow, nightly rhythm of pheromone release, female moths emit their sex pheromone blend in fast pulses via oscillatory exposure of their abdominal pheromone glands (Sasaki and Riddiford, 1984; Tumlinson et al., 1989; Riffell et al., 2008; Schendzielorz et al., 2015). Depending on wing fanning frequencies, Lepidoptera males detect intermittent changes of the pheromone/odor blend at specific ultradian frequencies, e.g., up to 25 Hz for *Bombyx mori* and up to 5 Hz for *Lymantria dispar* (Loudon and Koehl, 2000; Bau et al., 2005; Nakata et al., 2024). The males detect the pheromone blend with long trichoid sensilla on their antenna, each of which is innervated by two olfactory receptor neurons (ORNs). One of the two ORNs specifically detects bombykal (BAL), the main sex pheromone component, the other ORN responds to other components of the pheromone blend (Sanes and Hildebrand, 1976; Altner and Prillinger, 1980; Keil and Steinbrecht, 1984; Kaissling et al., 1989; Keil, 1989; Tumlinson et al., 1989). The properties of ORNs are controlled by their endogenous circadian clocks, which results in maximum sensitivity and fastest pheromone pulse tracking at night, during the hawkmoth's activity phase (Dolzer et al., 2003; Schuckel et al., 2007; Flecke and Stengl, 2009; Flecke et al., 2010).

Pheromone-sensitive ORNs of moths are outstandingly sensitive with a dynamic range that covers several log units of pheromone concentrations [e.g., *M. sexta*, ≥4 log units (Marion-Poll and Tobin, 1992; Dolzer et al., 2003); *B. mori*, 6–7 log units (Kaissling and Priesner, 1970)]. They are flux detectors, tuned to resolve fast changes in pheromone concentration (Baker and Vogt, 1988; Marion-Poll and Tobin, 1992; Kaissling, 1998). Their sensitivity and electrical response kinetics depend on stimulus strength and duration. Even brief exposure of several milliseconds to low concentrations of pheromone molecules results in prolonged alternation of the ORN spiking response (Dolzer et al., 2003). The molecular mechanisms, which are responsible for their exceptional sensitivity and which can be adjusted by zeitgeber time (ZT) and stimulus intensity, are poorly understood, and contradictory findings for insect pheromone/odor transduction cascades have been reported, even in the same species and for the same odorants (Nakagawa and Vosshall, 2009; Stengl, 2010, 2017; Stengl and Funk, 2013; Wicher and Miazzi, 2021).

Insect olfactory receptors (ORs) are seven-transmembrane (7TM) molecules with inverse topology and not related to the G-protein–coupled 7TM ORs of vertebrates (Clyne et al., 1999; Gao and Chess, 1999; Vosshall et al., 1999; Krieger et al., 2002, 2003; Benton et al., 2006; Lundin et al., 2007). In *Drosophila melanogaster*, ORs heteromerize with a distantly related but conserved inverse 7TM molecule, Or83b, the olfactory receptor coreceptor (Orco; Vosshall et al., 1999; Vosshall and Hansson, 2011). While an ionotropic mechanism was suggested for pheromone transduction in *B. mori* and both an ionotropic and mixed ionotropic and metabotropic transduction cascade for general odor detection in *D. melanogaster* (Sato et al., 2008; Wicher et al., 2008), no evidence for OR–Orco-dependent ionotropic pheromone transduction was found in *M. sexta* (Nolte et al., 2013, 2016). In *D. melanogaster*, OR–Orco complexes in heterologous expression systems constitute odorant-gated, nonspecific cation channels with slow-gating kinetics and a reversal potential ∼0 mV (Sato et al., 2008; Wicher et al., 2008). In contrast, in *M. sexta*, the first ion channel that activates after pheromone application is a transient Ca^2+^ channel with rapid kinetics that opens and closes in <100 ms. The resulting pheromone-dependent current resembles the inositol trisphosphate (IP_3_)- or diacyl glycerol (DAG)-dependent currents that are reminiscent of TRP ion channel family-dependent currents (Stengl et al., 1992; Stengl, 1993, 1994; Gawalek and Stengl, 2018; Dolzer et al., 2021). Thus, in hawkmoths, pheromone transduction was suggested to employ a metabotropic G-protein–coupled cascade that activates phospholipase Cβ (PLCβ), which allows strong signal amplification and a flexible, adjustable dynamic range (Stengl, 2010).

In contrast to most of the previous research on insect odor/pheromone transduction cascades, here, we used in vivo experiments in healthy *M. sexta* hawkmoths to challenge the hypothesis of metabotropic PLCβ -dependent pheromone transduction. We combined tip-recordings of pheromone-sensitive long trichoid sensilla with pharmacology in search for circadian modifications of pheromone transduction that are not directly mediated by light levels. We compared the ORN responses with BAL stimulation, where we applied synthetic BAL to the olfactory sensilla via an antenna-directed air stream, at the end of the hawkmoth's activity phase at ZT 1–3 to the response at the resting phase at ZT 9–11. The BAL-elicited spiking response consists of three consecutive components with distinct firing patterns and durations. Each of the components carries different information of the pheromone signal (Dolzer et al., 2003; Nolte et al., 2013, 2016). Changes in pheromone concentration are encoded only by the latency and spike frequency of the first component, the phasic response (∼10 spikes; Dolzer et al., 2003). Thus, a comprehensive data analysis of the distinct components of the pheromone/odor response is necessary because, while being conserved across insects, their distinct kinetics indicate different underlying mechanisms of insect olfaction (Kaissling, 1986, 1987; Dolzer et al., 2003; Nolte et al., 2013, 2016; Stengl and Funk, 2013; Stengl, 2017). Therefore, we carefully distinguished response parameters, both in terms of response component and ZT.

## Materials and Methods

### Animals

For all experiments, we used 2- to 3-d-old adult male *M. sexta* (Lepidoptera, Sphingidae) hawkmoths from the breeding and rearing colonies at the University of Kassel. Animals were raised on an artificial diet and kept under long-day conditions (L:D; 17:7 h) at 24–26°C and relative humidity of 40–60%. After pupation, males and females were separated, and males were housed isolated from pheromones. Animals were fed and raised as described in Gawalek and Stengl (2018). In brief, caterpillars were fed with an artificial diet modified after Bell and Joachim (1976); adult moths were fed with sucrose solution.

### Solutions

Salts for ringer solutions were purchased from Merck. Bacterial toxins, U73122 and U73343, were purchased from Sigma-Aldrich. All other chemicals were obtained from Sigma, unless stated otherwise. All substances were HPCL grade, unless noted otherwise.

Ringer solutions were prepared as described previously (Gawalek and Stengl, 2018). In brief, sensillum lymph ringer (SLR) contained the following (in mM), 172 KCl, 3 MgCl_2_, 1 CaCl_2_, 25 NaCl, 10 HEPES, and 22.5 glucose, pH 6.5, and adjusted to 475 mOsmol/kg with glucose. Hemolymph ringer (HLR) contained the following (in mM), 6.4 KCl, 12MgCl_2_, 1 CaCl_2_, 12 NaCl, 10 HEPES, and 340 glucose, pH 6.5, and adjusted to 450 mOsmol/kg with glucose.

Synthetic bombykal (BAL; E,Z-10,12-hexadecadienal; gift from J. H. Tumlison, Center for Medical, Agricultural and Veterinary Entomology) was prepared as stock solution of 100 µg/ml in *n*-hexane and stored at −80°C until use.

As general inhibitor of G-proteins, we used 10^−5^ M GDP-β-S (10^−2^ M stock solution dissolved in DMSO, Jena Bioscience) in SLR. For further discrimination between G-protein subunits, we used the following bacterial toxins. Pertussis toxin (pTox; 500 ng/500 µl solution in SLR, stored at 4°C until use) is a specific inhibitor of G_αo_, a subunit that usually inhibits adenylyl cyclase and therefore results in increases in cAMP levels (Mangmool and Kurose, 2011), in *D. melanogaster* (Hopkins et al., 1988). In insect antennae, pTox has been shown to inhibit the pheromone-dependent IP_3_ production (Boekhoff et al., 1990, 1993). Cholera toxin (cTox; purity ≥ 90%, 5 µg/500 µl solution in SLR, stored at 4°C until use) constitutively activates G_αs_, which activates adenylyl cyclase and leads to sustained increases in cAMP levels, also in insect antennae (Boekhoff et al., 1993; Deng et al., 2011). *Pasteurella multocida* toxin (pmTox; 500 ng/500 µl solution dissolved in SLR, stored at −20°C until use) activates G_αq_ and the G_αi/o_ subgroup in vertebrates, resulting in decreased cAMP and increased IP_3_ levels (Surguy et al., 2014; not yet tested in insects). For disruption of phospholipase C (PLC) signaling, we used the antagonist U73122 and its respective nonfunctional analog U73343 (both prepared as 10^−2^ M stock solution dissolved in DMSO and stored at −80°C until use), diluted to a final concentration of 10^−5^ M in SLR.

### Electrophysiology

To investigate the molecular mechanisms of the pheromone signal transduction cascade, we performed long-term (hours to days) tip-recordings of the ORNs of single antennal long trichoid sensilla (Kaissling, 1995) at room temperature (20–23°C). Animals were fixed in a custom-made holder with tape, and the antenna was immobilized with dental wax (Boxing Wax, Kerr) near the base. To record from ORNs, we slipped the recording electrode over the clipped tip of one long trichoid sensillum, and we inserted the reference electrode into the clipped flagellum close to the annulus where the recording electrode was located. Electrodes were moved with manual micromanipulators (MMJ, Märzhäuser Wetzlar; Leitz, Leica Microsystems). Electrodes were pulled from thin-walled borosilicate glass capillaries (OD, 1.5 mm; ID, 1.17 mm, Harvard Apparatus) with a tip opening that both sealed well around the sensillum and fit the inside of the antenna (∼1 µm). The opening of the cut flagellum around the reference electrode was sealed with electrode gel (GE Medical Systems Information Technology) to prevent dessiccation. The recording electrode was filled with SLR, and the reference electrode was filled with HLR. ORN activity was amplified 200-fold (custom build amplifier with 10^12^ Ω input impedance, or BA-03X, npi, or ELC-01MX, npi), low-pass filtered with a cutoff frequency of 1.3 kHz, digitized at 20 kHz with a Digidata (1200 or 1550A or 1550B, Molecular Devices), and saved for off-line analysis with pClamp 8 or 10 (Molecular Devices). We used pClamp to record the signal as high-pass filtered (150 Hz cutoff frequency) AC signal in addition to the unfiltered (DC) signal.

For BAL stimulation, 10 µl stock solution was loaded on filter paper (∼1 cm^2^), resulting in 100 ng BAL per filter paper. The solvent was allowed to evaporate and does not elicit responses from the BAL-sensitive ORN (Dolzer et al., 2003). Antenna-directed air streams for stimulation were set up according to previously described protocols (Nolte et al., 2013; Gawalek and Stengl, 2018). In brief, an odorless airstream was continuously blown over the antenna from a distance of ∼5 cm to adapt antennal mechanoreceptors. The airstream could be passed through a cartridge containing the BAL loaded filter paper via TTL switching of solenoid valves. BAL stimulation consisted of periodic application of a 50 ms pulse at an interstimulus interval of 5 min to avoid desensitization and habituation (Dolzer et al., 2003). Recordings with SLR in the recording electrode served as controls, either with or without the addition of 0.1% DMSO, as specified. The addition of 0.1% DMSO to the SLR did not alter the recorded BAL response. Activators, inhibitors, or toxins were added to the SLR of the recording electrode.

To search for ZT-dependent effects, we recorded during the animal's late activity phase (ZT 1–3) and in the middle of its resting phase (ZT 9–11). In experiments involving G-protein or PLC signaling disruption, we recorded from different animals at the different ZTs and different interference conditions. Recording and periodic stimulation usually lasted for 120 min, with a few exceptions in the control recordings, which only lasted for ∼70 min. In the toxin experiments, we did paired recordings of the same animal at the same ZTs on two consecutive days. For these experiments, we always filled the recording electrode with toxin SLR, obtained the control recordings on Day 1 immediately after attaching the electrode but before the toxins had any effect, and recorded on the same ZT on Day 2 without removing the electrodes in between. Here, we stimulated three times in the control recording and six times in the toxin recording.

### Tissue collection, RNA extraction, and cDNA synthesis

We collected antennal tissue from isolated cultures of 2-d-old males. At each ZT 1, ZT 9, and ZT 17, we collected three samples with eight antennae per sample. All samples were immediately frozen in liquid nitrogen and stored at −80°C for RNA extraction. Total RNA was isolated using TRIzol Reagent (Invitrogen) according to the manufacturer's protocol. The quality of the RNA was assessed by gel electrophoresis. The concentration and purity of RNA were detected using NanoDrop ND-1000 spectrophotometer (Thermo Fisher Scientific). OD_260/280_ ratios of all samples were 1.8–2.0. The first-strand complementary DNA (cDNA) was synthesized with 1 μg of total RNA for each sample using the PrimeScript RT reagent Kit with gDNA Erase (Takara Bio) according to the manufacturer's instructions and stored at −20°C until use.

### Phylogenetic analysis of *PLCβ* from *M. sexta* and other species

Since the *PLCβ* gene is not annotated clearly in the genome of *M. sexta*, we utilized the coding sequences (cds) of the *PLCβ* genes from *D. melanogaster* and *B. mori*. We identified two highly similar genes in *M. sexta* as candidate *PLCβ1* (*Plc21C*; gene ID: LOC115440592) and *PLCβ4* (*norpA*; gene ID: LOC115451385) with BLAST analysis in the NCBI database. We downloaded the cds of these candidate genes and the *PLC-β* genes of other insects, *Homo sapiens* and *Mus musculus*. We translated the cds into protein sequences and performed sequence alignments using MEGA X (Kumar et al., 2018). The phylogenetic tree was constructed using the maximum likelihood method and Tamura-Nei model (Tamura and Nei, 1993), and node support was evaluated with 1,000 bootstrap replicates.

### Real-time quantitative polymerase chain reaction (qPCR)

qPCR was conducted to determine the expression of target genes (Table 1) at ZT 1, ZT 9, and ZT 17 of hawkmoth antennae with TB Green Premix Ex Taq II (Tli RNase H Plus, Takara Bio). Primers for each target gene were designed using the Primer-BLAST online program of NCBI (Table 1). *Ribosomal Protein S13* (*RPS13*; Fenske et al., 2018) and *Glyceraldehyde-3-Phosphate Dehydrogenase* (*G3PDH*; Mészáros and Morton, 1996; Adamo et al., 2016) were chosen as the reference genes to normalize the relative expression levels of each target gene. Agar gel electrophoresis, sequencing of PCR fragments, and subsequent qPCR showed that the expression levels of the reference genes in the antennae were stable at ZT 1, ZT 9, and ZT 17. In this study, all primers were used to amplify the target genes via PCR with cDNA. Following amplification, the PCR products were resolved by gel electrophoresis. Target bands were excised and purified using NucleoSpin Gel and PCR Clean-Up Kit (Macherey-Nagel). The purified DNA was subsequently sequenced using the Sanger method to verify the specificity of the amplified fragments.

**Table 1.** Forward and reverse primer sequences for target and reference genes. Asterisks indicate candidate reference genes.

Gene	Primer sequence (5′-3′)	Size (bp)	Reference
*timeless*	F: TTAAGCCGACCGTAGTGCTG	120	-
	R: CGTCTTCCGTCCATGTGTCT		
*G_αo_*	F: AAGCTGCTGCTCCTTGGTGCT	147	-
	R: GGCGACGAGCGATTGAATAGTG		
*G_αq_*	F: CATTCACGGGTCGGGTTACA	151	-
	R: CTCCGCCTTTTCTACGTTGG		
*G_αs_*	F: AGTCGACCATCGTGAAGCAG	126	-
	R: TTGCACCGGTGATCGTAAGT		
*PLCβ1*	F: CGACACCATAAGCCAGTCCA	115	-
	R: CGGTTTGCCGTCGGTTAAAT		
*PLCβ4*	F: GATATGGATCAGCCCCTCGC	137	-
	R: AGTTCTACGCACCTGCATCC		
RPS13*	F: GTCTTGCCCCTGACCTACCT	173	(Fenske et al., 2018)
	R: TGGCAGCACACTCTTTGTCT		
G3PDH*	F: CGATTAAGGAACTTGAGGACG	232	(Mészáros and Morton, 1996; Adamo et al., 2016)
	R: ATAAGGAAGCGGATGCAAGG		

The qPCR reaction program was 95°C for 30 s, followed by 40 cycles of 95°C for 5 s and 60°C for 30 s. For each gene and biological repeat there were three technical replicates. The amplification efficiency of all primers was 90–110%. The relative expression levels of these target genes were calculated according to the 2^−ΔΔCt^ method (Livak and Schmittgen, 2001).

### Data analysis

**Figure 1.**
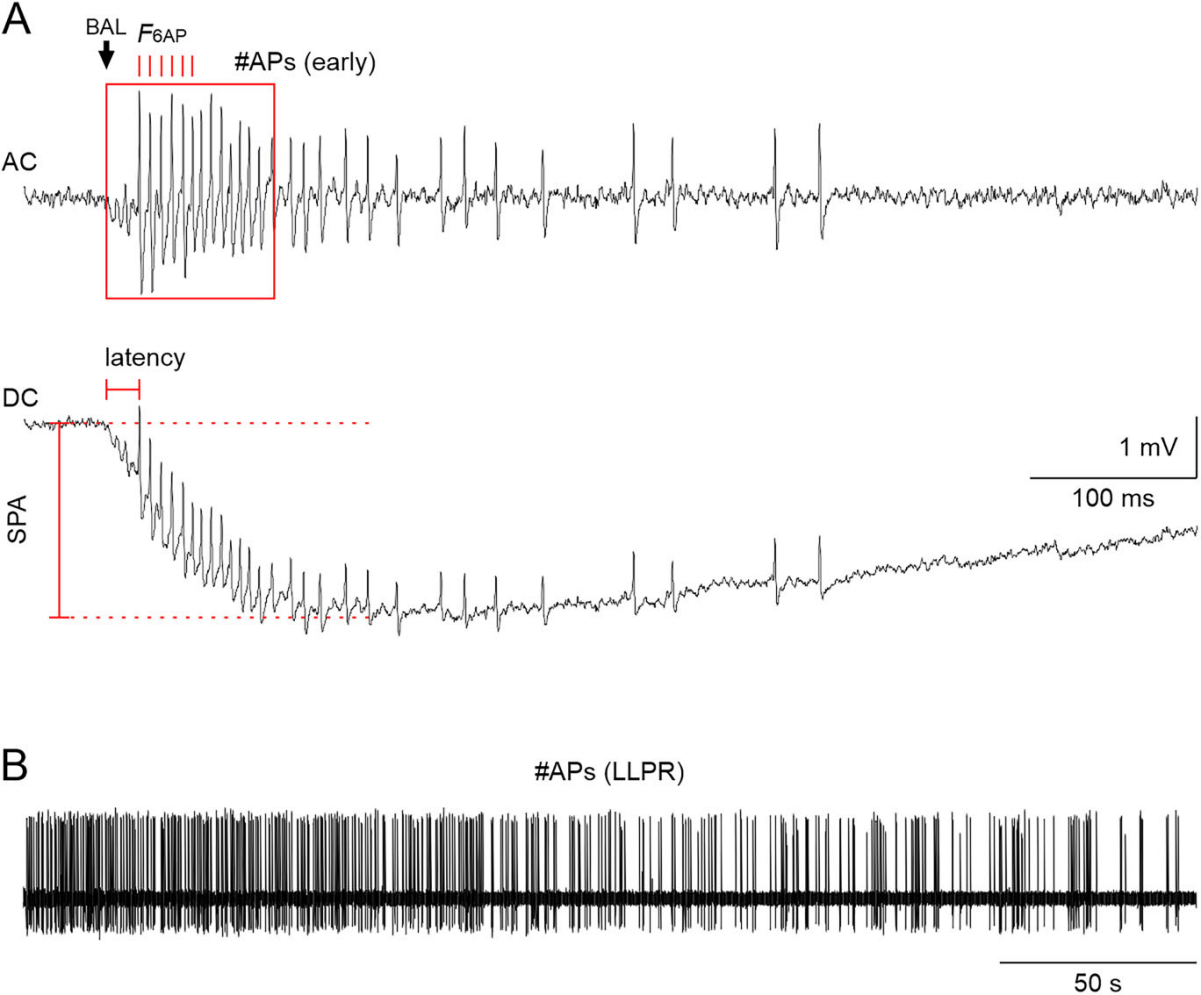
Quantification of the three phases of the pheromone response of hawkmoth long trichoid sensilla. The BAL-elicited spiking response consists of three consecutive spiking patterns (3 components) with distinct kinetics but variable duration (1) a phasic, high-frequency spiking response that lasts <100 ms (***A***), (2) a tonic spiking response with lower and more variable spiking frequency that lasts >100 ms (***A***), and (3) a late long-lasting spiking response (LLPR) that lasts seconds to minutes (***B***) (Dolzer et al., 2003; Nolte et al., 2016). ***A***, High-pass filtered (AC, top trace) and unfiltered (DC, bottom trace) recording of the sensillum potential with APs of the phasic–tonic ORN response to BAL. BAL stimulus: 50 ms, 10 µl of 0.1 mg/ml on 1 cm^2^ filter paper; arrow: start of the BAL response. For the phasic response, we calculated the average instantaneous AP frequency of the first six APs (*F*_6AP_, red ticks, top trace) of the BAL response. A combination of both phasic and part of the tonic response was evaluated as the number of APs in a 100 ms window starting at the onset of the BAL response [#APs (early), red box, top trace]. Latency (red horizontal marking, bottom trace) is the time from the beginning of the BAL response to the first AP. SPA is the amplitude from the baseline voltage before BAL stimulation to the negative peak (red vertical marking, bottom trace) (***B***) High-pass filtered recording of the LLPR. The LLPR was evaluated as the number of APs [#APs (LLPR)] in the 295 s before the next BAL stimulus, excluding the first 5 s after the BAL stimulus.

For evaluation of pheromone responses, both the unfiltered (DC) and high-pass filtered signal (AC) were examined. Spikes were detected by a threshold search on the AC trace. The following pheromone response parameters of the ORN spike train were evaluated during the first second after BAL stimulation (Fig. 1*A*): latency of the first spike relative to the beginning of the DC pheromone response, phasic spike frequency as the average instantaneous frequency of the first six spikes (*F*_6AP_), the number of spikes in a 1 s window (general G-protein and PLC disruption experiments), or 100 ms window [toxin experiments; #APs (early)], and sensillum potential amplitude (SPA). The SPA was measured as the maximal deflection of the transepithelial potential following the BAL stimulation. Furthermore, for analysis of response kinetics, all action potentials (APs) within the first second after BAL stimulation were counted and binned in 10 ms bins. For the late long-lasting pheromone response (LLPR) that persists over several minutes after pheromone stimulation, we counted the number of spikes in the 295 s before the next BAL stimulus (Fig. 1*B*).

**Figure 2.**
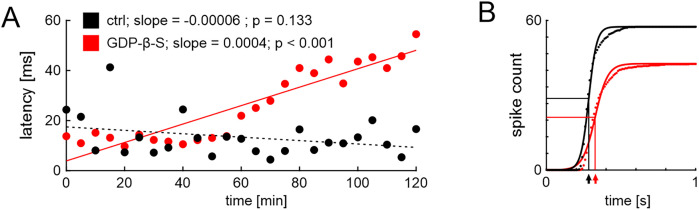
Illustration of the linear fit analysis of pheromone responses. ***A***, BAL stimuli were applied every 5 min in the 120-min-long tip-recordings of hawkmoth trichoid sensilla. For each animal and experimental condition [here, one control (black) and one GDP-β-S (red) animal], we generated a linear regression model for time and respective BAL response parameters (here, latency to the first AP after the onset of the BAL response) and used a *t* test to determine whether the slope of the fit significantly differed from zero (solid line) or not (dashed line). ***B***, To quantify the response kinetics to BAL stimulation, we binned the data of the first second after the BAL stimulus in 10 ms bins across all BAL stimulations for each animal. Each resulting cumulative histogram (dotted line) was fitted with a sigmoidal function (solid line) for quantification that yielded three fit parameters: maximum spike number (AP_max_), time of the midpoint of the sigmoid (t_1/2_; indicated by arrows at the *x*-axis), and slope of the midpoint (k). Depicted are examples of two animals.

In experiments involving G-protein or PLC signaling disruption, the time course of the responses to repeated stimulation varied across animals, presumably due to individual differences in the reaction to DMSO application in vivo or differences between individual sensilla. First effects were usually observed within 10–30 min after placement of the electrodes, but due to the in vivo recording method, the final concentration of the chemicals in the sensillum lymph was unknown. Therefore, we used linear fits to describe the change of each response parameter over the 120 min of stimulation. The slopes of the fits were used for quantification (Fig. 2*A*). Slopes that were not significantly different from zero (no significant change of the response over time, *t* test) were set to zero.

To quantify the changes of the kinetics of the spiking response to BAL stimulation, we created cumulative histograms of the binned data and fitted those histograms with the sigmoidal function as follows:

$$\mathrm{AP}(t)=\frac{AP_{\max}}{1+\exp\frac{t-t_{1/2}}{k}},$$

where AP_max_ is the cumulative number of spikes within 1 s after stimulation, *t*_1/2_ is the time of the midpoint, and *k* is the slope factor (Fig. 2*B*). The fit parameters were used for quantification.

### Statistics

Data were tested for normal distribution with the Shapiro–Wilk test and for equal variance with Levene's test. We used one-way ANOVA or repeated-measure (RM) ANOVA for normally distributed data with equal variance as indicated. If data were not normally distributed, we log-transformed the data or used ANOVA on ranks if transformation did not result in a normal distribution. For pairwise comparisons, we used Tukey's post hoc test for normally distributed and Dunn's post hoc test for non-normally distributed data. All tests were done in SigmaPlot (version 12, Systat Software).

## Results

The response of antennal pheromone-sensitive trichoid sensilla to BAL stimulation was recorded in vivo in restrained, intact male hawkmoths at two ZTs: at the end of the activity phase (ZT 1–3) and in the middle of the resting phase (ZT 9–11). Pharmacology was used to determine whether hawkmoth ORNs comprise G-protein–coupled pheromone receptors that activate PLCβ and whether this pathway is under the control of an ORN-based endogenous circadian clock to maximize sensitivity and temporal resolution of pheromone detection during the activity phase.

The pheromone response of ORNs consists of three components. The first component is fast and phasic and comprises <10 spikes (APs) which encode BAL concentration changes (Dolzer et al., 2003). It is characterized by regular, high-frequency spiking in a time window of ≤ 100 ms. The second component is tonic spiking at a lower and more variable frequency, which does not encode pheromone concentration changes but encodes signal duration within a limited range. It typically lasts several hundreds of milliseconds. This is followed by a pause and then the third component, the spiking of the LLPR, which can last seconds to minutes. The LLPR encodes neither quantity nor duration of the pheromone signal. It is distinct from the spontaneous activity before pheromone application and is hypothesized to encode a memory trace of the previous pheromone signal (Stengl, 2010).

### General inhibition of G-proteins reduced the spike frequency and increased response latency of the phasic component of the BAL response during the activity phase but not during rest

To search for G-protein coupling in the pheromone transduction cascade, 10 µM GDP-β-S, an indiscriminative inhibitor of G-proteins, were infused via the tip-recording electrode into the pheromone-sensitive trichoid sensillum. The ORNs’ neural responses to BAL stimulation were recorded with a nonadapting, periodic stimulation protocol (Dolzer et al., 2003) at two different ZTs in control and with GDP-β-S.

**Figure 3.**
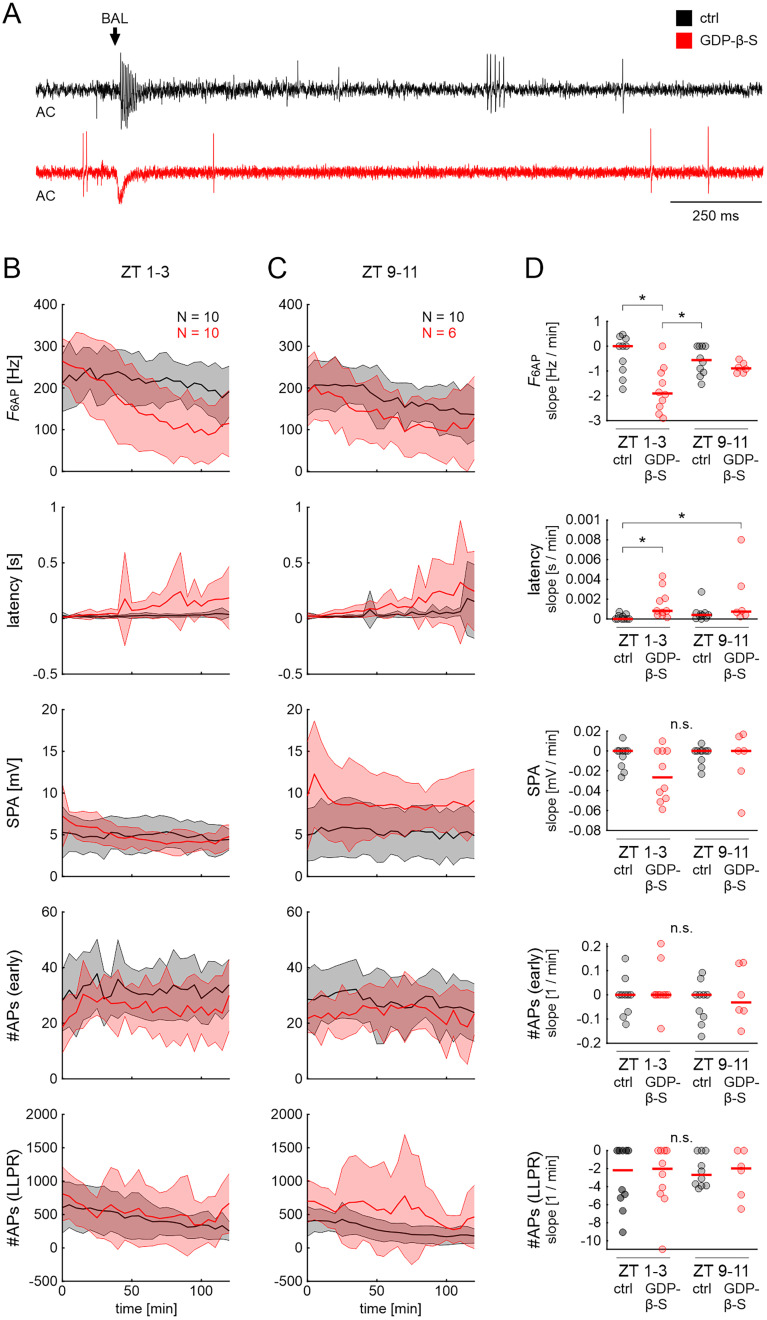
Inhibition of G-protein signaling of pheromone-sensitive ORNs by GDP-β-S decreased the phasic BAL response and increased response latency during the hawkmoth's activity phase. ***A***, Examples of high-pass filtered tip-recordings (AC) of ORN responses in control (ctrl, recording solution + DMSO, black) and with the G-protein antagonist GDP-β-S (dissolved in SLR + DMSO, red) at ZT 1–3, 100 min after the start of the recording. Arrow indicates the onset of BAL response. Response parameters (Fig. 1) during the moth's late activity phase (ZT 1–3; ***B***) and at rest (ZT 9–11; ***C***) in control and GDP-β-S. Data are shown as mean (line) ± standard deviation (shaded area). ***D***, One-way ANOVA results with appropriate post hoc test for multiple comparisons (*α* = 0.05) for the slopes of BAL response parameters (see Materials and Methods and Fig. 2*A*). *F*_6AP_ slopes decreased significantly, and latency slopes increased significantly in GDP-β-S compared with control at the activity phase (ZT 1–3). No other parameters showed significant differences compared with control at either ZT. Dots show data for individual experiments; red lines indicate the mean. Raw data are provided in Extended Data Figure 3-1.

**Figure 4.**
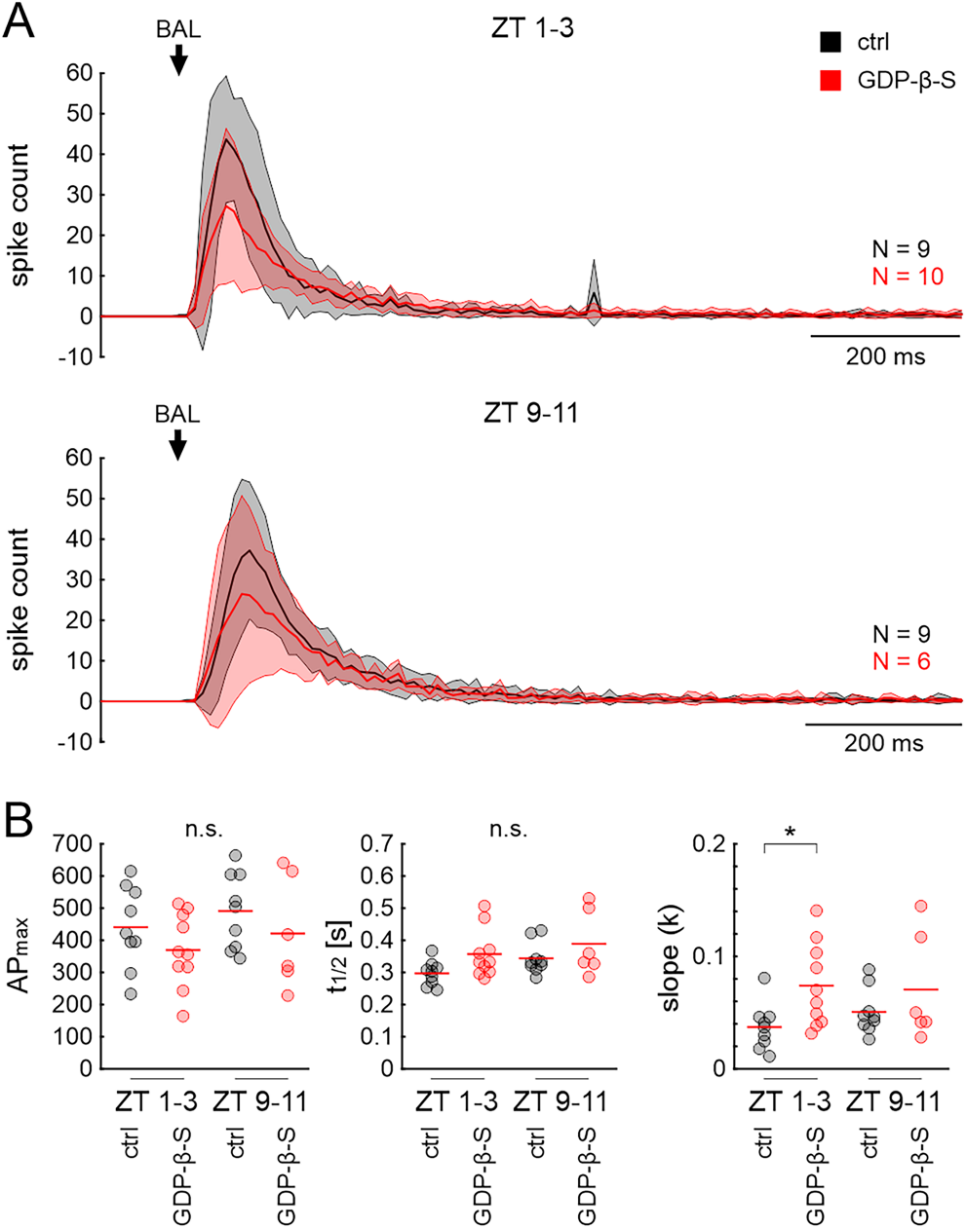
Inhibition of G-protein signaling changed kinetics of the ORN response to BAL stimulation during the activity phase. ***A***, Peristimulus time histograms (PTSH; 10 ms bins) of the first second of the ORN response to BAL stimulation in control (black) and in GDP-β-S (red). Data are shown as mean (line) ± standard deviation (shaded area). Arrow: onset of BAL response. ***B***, Fit parameters [total number of spikes in 1 s after onset of the BAL response (AP_max_), time of the sigmoid midpoint (t_1/2_), and slope (k) of the midpoint] of sigmoidal fits to the cumulative spike histograms (see Eq. 1, Materials and Methods, and Fig. 2*B*). One-way ANOVA with appropriate post hoc test (*α* = 0.05) revealed a significantly steeper slope (*k* closer to zero) in control compared with GDP-β-S during the activity phase (ZT 1–3). Steeper slopes indicate faster rise and fall times of the spike count in the PTSHs. The total number of spikes and sigmoid midpoint were not significantly different. Dots show fit parameter values for individual experiments; red lines indicate the mean. Raw data are provided in Extended Data Figure 4-1.

During the activity phase, blocking of G-protein–coupled processes reduced the frequency of the phasic response component (*F*_6AP_), while the latency increased (Fig. 3*A*,*B*,*D*; ANOVA: Table 2; raw data: Extended Data Fig. 3-1). In contrast, GDP-β-S had no significant effect on *F*_6AP_ or latency during the resting phase (Fig. 3*B*–*D*; ANOVA: Table 2; raw data: Extended Data Fig. 3-1). However, response variability across animals increased in GDP-β-S at both ZTs over time, as indicated by the large increase in standard deviation (Fig. 3*B*,*C*). In addition, the kinetics of the response, i.e., rise and fall times to the maximum spike rate, were diminished with GDP-β-S during the activity phase but not at rest, as indicated by the larger slope factor *k* of the sigmoidal fits (Fig. 4; ANOVA: Table 3; raw data: Extended Data Fig. 4-1). GDP-β-S application did not have significant effects on any of the other response parameters at either ZT.

10.1523/ENEURO.0376-24.2024.f3-1 Figure 3-1 All data for the analysis shown in Figure 3. The excel file contains five sheets: one with the slopes and four with the raw data for the four combinations of ZT and experimental conditions from which the slopes were fitted. “slopes” contains the two datasets at ZT 1-3 and ZT 9-11. In each dataset, the first column is the unique experiment ID, the second column denotes the experimental condition, and the following columns give the slope for the five response parameters. In the other four sheets, the first column denotes the stimulation time in minutes after the electrodes were attached. Here, data are organized by experiment ID and show the value of the respective response parameter for each stimulation time point. “NaN” indicates those trials where we could not extract the relevant parameters from the recording. Download Figure 3-1, XLS file.

10.1523/ENEURO.0376-24.2024.f4-1 Figure 4-1 All data for the analysis shown in Figure 4. Each dataset is a combination of ZT and experimental condition. For each dataset, the first column denotes the experiment ID, and the following three columns the respective fit parameters. Download Figure 4-1, XLS file.

**Table 2.** One-way ANOVA results for BAL response parameters in control and GDP-β-S

*N*	Parameter	Normality	Variance	Test statistic	df	Res.	*p*
10	*F*_6AP_	Pass	Pass	*F* = 7.492	3	32	**<0.001**
10	latency	Fail		*H* = 13.975	3		**0.003**
10	SPA	Pass	Fail	*H* = 3.497	3		0.321
10	#APs (early)	Pass	Pass	*F* = 0.546	3	32	0.654
10	#APs (LLPR)	Fail		*H* = 0.203	3		0.977

If tests for normality (Shapiro–Wilk) or equal variance (Levene) failed, results are for ANOVA on ranks. The four groups are ctrl ZT 1–3, GDP-β-S ZT 1–3, ctrl ZT 9–11, and GDP-β-S ZT 9–11. *p* values smaller than *α* = 0.05 are printed in bold. *N*, number of animals; df, degrees of freedom; res: residual.

**Table 3.** One-way ANOVA results for the fit parameters of the BAL response kinetics (Eq. 1)

*N*	Parameter	Normality	Variance	Test statistic	df	res.	*p*
10	AP_max_	Pass	Pass	*F* = 1.414	3	30	0.258
10	*t*_1/2_	Fail		*H* = 7.243	3		0.065
10	Log(slope)	Pass	Pass	*F* = 3.212	3	30	**0.037**

Data for slope were log-transformed to ensure normal distribution. If tests for normality (Shapiro–Wilk) or equal variance (Levene) failed, results are for ANOVA on ranks. The four groups are ctrl ZT 1–3, GDP-β-S ZT 1–3, ctrl ZT 9–11, and GDP-β-S ZT 9–11. *p* values smaller than *α* = 0.05 are printed in bold. *N*, number of animals; df, degrees of freedom; res, residual.

In conclusion, only the first component of the BAL response, which encodes stimulus concentration (*F*_6AP_, latency), appears to be under the control of G-proteins. This metabotropic signaling changed with ZT.

### Inhibition of PLC reduced the spike frequency of the phasic component of the BAL response at both ZTs while increasing response latency and decreasing SPA during the activity phase

**Figure 5.**
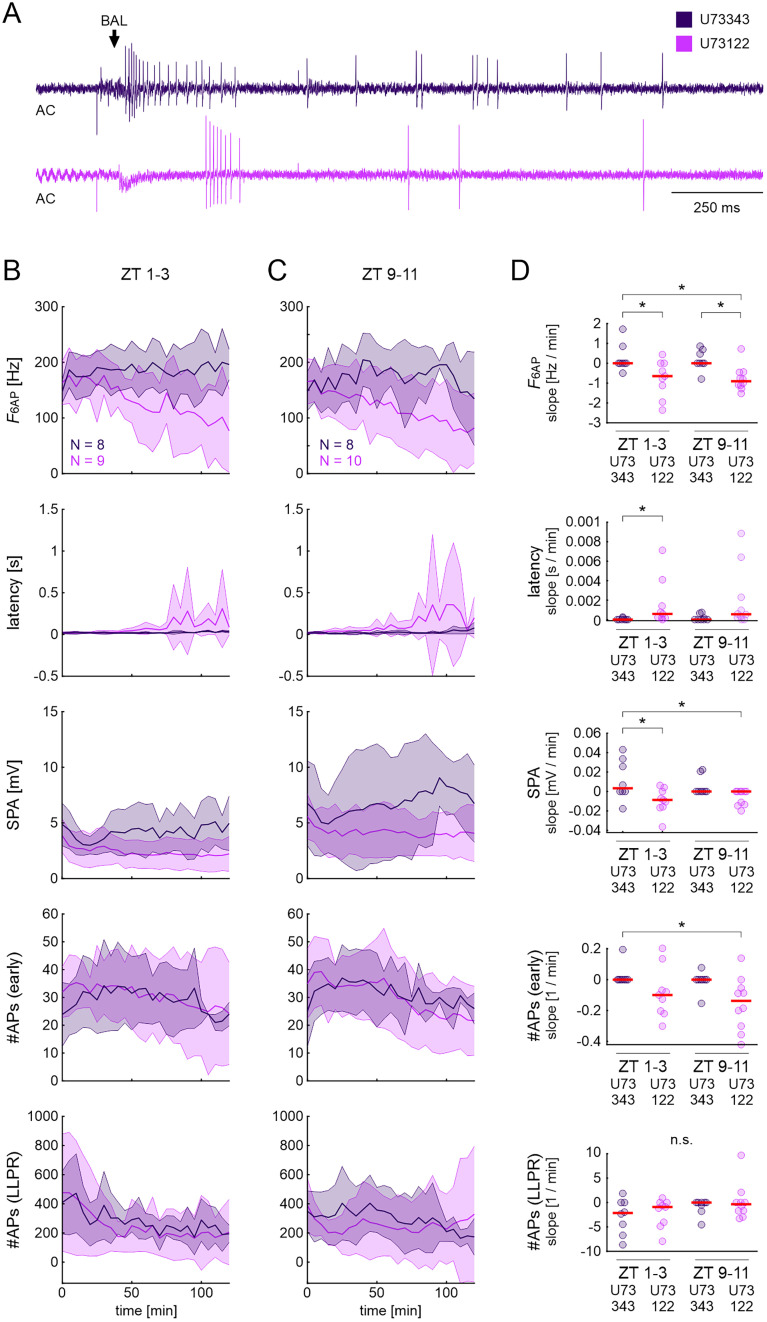
Inhibition of PLC of pheromone-sensitive ORNs with U73122 decreased the phasic BAL responses at both ZTs while decreasing SPA and increasing response latency only at ZT 1–3. ***A***, Example high-pass filtered (AC) tip-recordings of ORN responses in control (U73343, dark purple) and in PLC inhibitor (U73122, light purple) at ZT 1–3, 100 min. The arrow indicates the onset of the BAL response. Response parameters (Fig. 1) during the activity phase (ZT 1–3; ***B***) and at rest (ZT 9–11; ***C***) in control and U73122. Data are shown as mean (lines) ± standard deviation (shaded areas). ***D***, One-way ANOVA results with appropriate post hoc test for multiple comparisons (*α* = 0.05) for the slopes of BAL response parameters (see Materials and Methods and Fig. 2*A*). Compared with controls, *F*_6AP_ slopes decreased at both ZTs in U73122, but SPA and latency slopes increased only at the activity phase (ZT 1–3). Other BAL response parameters showed no significant differences between control and U73122 at either ZT. Dots show data of individual experiments; red lines indicate the mean. Raw data are provided in Extended Data Figure 5-1.

In a complementary experiment, the PLC inhibitor U73122 was infused via the tip-recording electrode during the same ZTs as in the experiment above, where G-protein action was blocked. Infusion of U73343, a structurally similar but very weak inhibitor of PLC, served as the control. Overall, results were comparable with those obtained in GDP-β-S with some ZT-dependent differences (Fig. 5; ANOVA: Table 4; raw data: Extended Data Fig. 5-1).

10.1523/ENEURO.0376-24.2024.f5-1 Figure 5-1 All data for the analysis shown in Figure 5. The excel file contains five sheets: one with the slopes and four with the raw data for the four combinations of ZT and experimental conditions from which the slopes were fitted. “slopes” contains the two datasets at ZT 1-3 and ZT 9-11. In each dataset, the first column is the unique experiment ID, the second column denotes the experimental condition, and the following columns give the slope for the five response parameters. In the other four sheets, the first column denotes the stimulation time in minutes after the electrodes were attached. Here, data are organized by experiment ID and show the value of the respective response parameter for each stimulation time point. “NaN” indicates those trials where we could not extract the relevant parameters from the recording. Download Figure 5-1, XLS file.

**Table 4.** One-way ANOVA results for BAL response parameters in control (U73343) and U73122

*N*	Parameter	Normality	Variance	Test statistic	df	res.	*p*
10	*F*_6AP_	Pass	Pass	*F* = 5.447	3	31	**0.004**
10	Latency	Fail		*H* = 12.187	3		**0.007**
10	SPA	Pass	Pass	*F* = 4.493	3	31	**0.010**
10	#APs (early)	Pass	Fail	*H* = 10.327	3		**0.016**
10	#APs (LLPR)	Pass	Pass	*F* = 1.558	3	31	0.219

If tests for normality (Shapiro–Wilk) or equal variance (Levene) failed, results are for ANOVA on ranks. Groups are ctrl ZT 1-3, U73122 ZT 1-3, ctrl ZT 9-11, and U73122 ZT 9-11. *p* values smaller than *α* = 0.05 are printed in bold. *N*, number of animals; df, degrees of freedom; res, residual.

During the activity phase, the inhibition of PLC significantly reduced *F*_6AP_, increased latency, and reduced the SPA over time (Fig. 5*B*,*D*). In addition, the inhibition of PLC significantly reduced *F*_6AP_ over time during the resting phase (Fig. 5*C*,*D*). All other response parameters did not show significant differences at either ZT. Similarly to the experiments with GDP-β-S, response variability across animals increased in U73122 at both ZTs over time, as indicated by the large increase in standard deviation (Fig. 5*B*,*C*).

In conclusion, the inhibition of PLC, a downstream target of G-proteins, affected mainly the first component of the BAL response. Most of the effects were observed during the activity phase of the animals.

### Toxins targeting G_αo_ and G_αs_ reduced the spike frequency and increased the response latency of the phasic component of the BAL response during the activity phase

Thus far, the results indicated that G-protein signaling and activation of PLC were involved in the hawkmoth pheromone transduction cascade. Typically, PLC is activated by the αq subunit of G-proteins and increases IP_3_ levels. Therefore, the bacterial toxins cTox, pTox, and pmTox were applied to specifically activate or inhibit different G_α_ subunits, which interferes with cAMP and IP_3_ levels (see Materials and Methods). These experiments were done only during the hawkmoth's activity phase at ZT 1–3 because G-protein and PLC inhibition in the previous experiments were effective at those times. As with the chemicals before, toxins were applied to the sensillum via the recording electrode.

**Figure 6.**
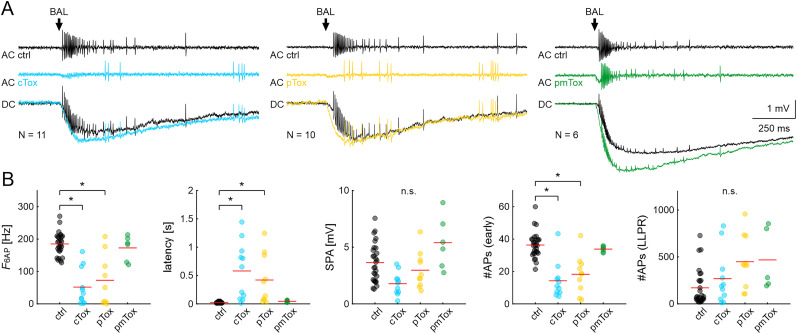
Responses to BAL stimulation at ZT 1–3 were affected by toxins that target G_α__s_ or G_α__o_ subunits. ***A***, Examples of tip-recordings from ORNs with BAL stimulation in control (black), with cTox (blue), pTox (yellow), and pmTox (green); repeated measures of one animal shown for each toxin. AC: high-pass filtered recording to highlight AP response; DC: unfiltered SPA with APs; Arrow: onset of the BAL response. ***B***, Quantification with one-way RM ANOVA with appropriate post hoc test for multiple comparisons (*α* = 0.05) of the same parameters as in Figure 3 and Figure 5. During the activity phase (ZT 1–3), the frequency of the phasic BAL responses (F_6AP_) and the number of APs during the first 100 ms of the response [#APs (early)] decreased in cTox (blue; sustained activation of G_α__s_) and pTox (yellow; inhibition of G_α__o_), while response latency increased significantly. The effect of pmTox (green; constitutive activation of G_α12/13_, G_αi_, and G_αq_) was not different from control. Raw data are provided in Extended Data Figure 6-1.

Both cTox and pTox application resulted in significant differences in the first and second component of the BAL response that were similar to the results obtained with GDP-β-S and U73122: activation of G_αs_ and inhibition of G_αo_ both decreased *F*_6AP_ and increased latency significantly (Fig. 6; ANOVA: Table 5; raw data: Extended Data Fig. 6-1). In addition, these toxins significantly reduced the number of spikes in the first and second component of the response [#APs (early)]. SPA and #APs (LLPR) were not altered. The application of pmTox did not have any significant effects (Fig. 6*B*).

10.1523/ENEURO.0376-24.2024.f6-1 Figure 6-1 All data for the analysis shown in Figure 6. The excel file contains three sheets, one for each toxin condition. Data are grouped by experiment ID. The first column contains the stimulation time points. Subsequent columns contain the respective response parameter at that time point. Experiments were paired with control recordings on day 1 and toxin recordings on day 2. “NaN” indicates those trials where we could not extract the relevant parameters from the recording. Download Figure 6-1, XLS file.

**Table 5.** One-way RM ANOVA results for BAL response parameters in control and bacterial toxins at ZT 1–3

*N*	Parameter	Normality	Variance	Test statistic	df	res.	*p*
28	*F*_6AP_	Pass	Pass	*F* = 26.611	3	23	**<0.001**
28	Log(latency)	Pass	Pass	*F* = 26.606	3	25	**<0.001**
28	SPA	Pass	Pass	*F* = 1.996	3	24	0.142
28	#APs (early)	Pass	Pass	*F* = 22.252	3	24	**<0.001**
28	#APs (LLPR)	Pass	Pass	*F* = 3.726	3	23	**0.026**

Values for latency were log-transformed to ensure equal variance. Groups are ctrl, pTox, cTox, pmTox. *p* values smaller than *α* = 0.05 are printed in bold. *N*, number of animals; df, degrees of freedom; res, residual.

In conclusion, toxins that are known to interfere with G-protein–dependent activation/disinhibition of adenylyl cyclase affected the BAL transduction in hawkmoths in the same way as PLC inhibition. Since pmTox did not result in significant changes of the BAL response, it remains to be studied whether pmTox affects any G-proteins in hawkmoths. The application of the toxins that increase cAMP concentration replicated the results that we obtained from G-protein and PLC inhibition.

### Expression levels of PLCβ4 peaked during the activity phase

**Figure 7.**
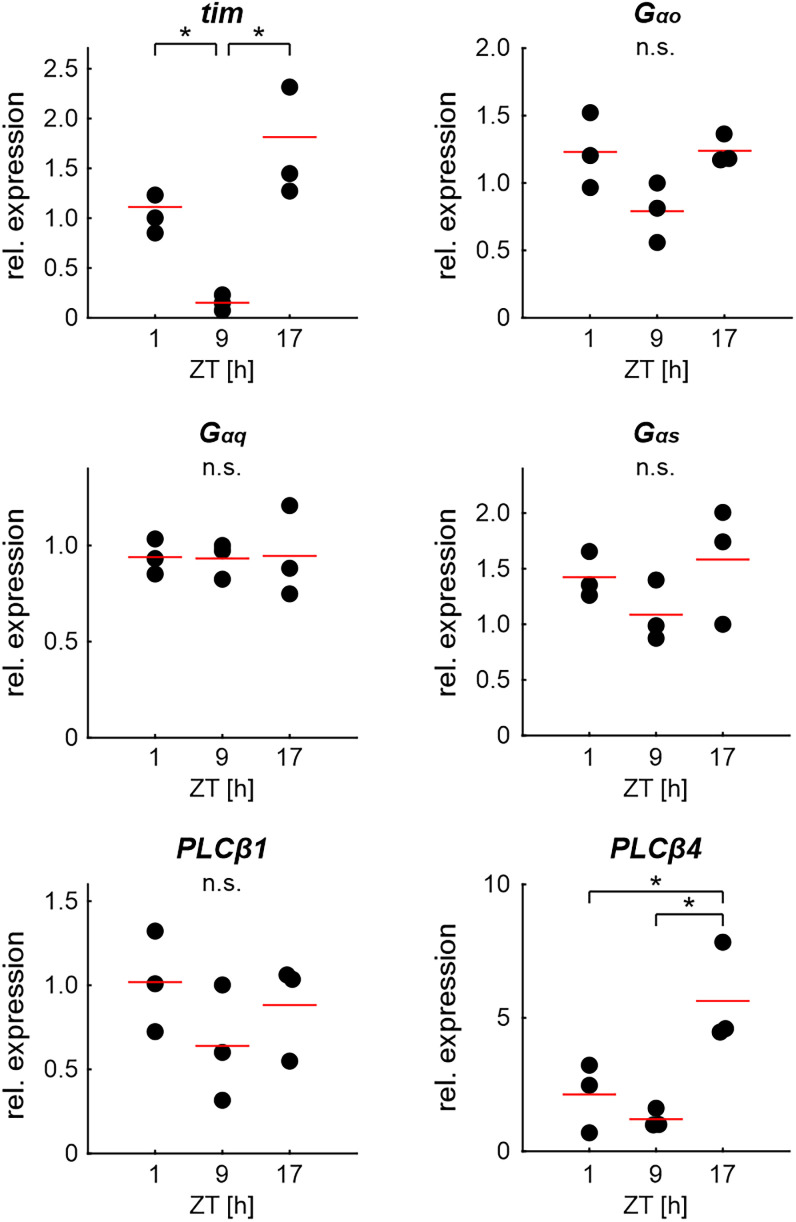
Relative expression levels of mRNA for G-proteins, PLCβ, and the circadian clock protein timeless (*tim*) in male hawkmoth antenna at different ZTs. qPCR revealed that mRNA levels of *PLCβ4* peaked significantly at the beginning of the activity phase at ZT 17. G_αo_, G_αq_, G_αs_, and *PLCβ1* did not change throughout the day. The mRNA of the cycling circadian clock protein timeless (*tim*) served as the positive control. Dots indicate values for biological replicates; each biological replicate contains extracts from eight antennae and three technical repeats. Red lines depict the mean. One-way ANOVA with appropriate post hoc test for pairwise comparisons (*α* = 0.05). The phylogenetic tree for PLCβ is provided in Extended Data Figure 7-1. Nucleotide sequences of the genes are provided in Extended Data Figure 7-2.

During the activity phase of the hawkmoth, but not during the resting phase, the disruption of G-proteins and PLC affected the pheromone response parameters that encode BAL concentration. Therefore, we examined whether these effects were caused by circadian changes in the antennal expression levels of G_αo_ or G_αq_, which activate PLCβ (increased IP_3_ levels), or G_αs_, which activates adenylyl cyclase (increased cAMP levels). The expression levels of G_α_ subunits and PLCβ, which we found in the transcriptome of male hawkmoth antennae, were quantified with qPCR (Fig. 7). Phylogenetic analysis clustered the putative PLCβ candidate transcripts with those of *B. mori* (Extended Data Fig. 7-1; the nucleotide sequences of the genes are listed in Extended Data Fig. 7-2). Expression of the circadian clock gene *timeless* (*tim*) served as the positive control. Only *tim* and PLCβ4 (*norpA*) showed significantly different expression levels throughout the day, with higher expression levels during the activity phase (Fig. 7; Table 6). The expression of G_α_ subunits and PLCβ1 did not change.

10.1523/ENEURO.0376-24.2024.f7-1 Figure 7-1 The phylogenetic tree was constructed using the maximum likelihood method, and node support was evaluated with 1000 bootstrap replicates. Bootstrap values > 67 are shown. Black boxes and black circles represent candidate PLCβ1 and PLCβ4 of *M. sexta* (Gene IDs: LOC115440592 and LOC115451385), respectively. Sequence information is detailed in Extended Data Figure 7-2. Download Figure 7-1, TIF file.

10.1523/ENEURO.0376-24.2024.f7-2 Figure 7-2 Nucleotide sequences for the genes used in the phylogenetic analysis. Download Figure 7-2, XLS file.

**Table 6.** One-way ANOVA results for gene expression levels at ZT 1, ZT 9, and ZT 17

*N*	Gene	Normality	Variance	Test statistic	df	res.	*p*
3	*tim*	Pass	Pass	*F* = 14.889	2	6	**0.005**
3	G_αo_	Pass	Pass	*F* = 4.276	2	6	0.070
3	G_αq_	Pass	Pass	*F* = 0.005	2	6	0.995
3	G_αs_	Pass	Fail	*F* = 1.479	2	6	0.301
3	PLCβ1	Pass	Pass	*F* = 1.137	2	6	0.381
3	PLCβ4	Pass	Pass	*F* = 9.001	2	6	**0.016**

*p* values smaller than *α* = 0.05 are printed in bold. *N*, number of animals; df, degrees of freedom; res, residual.

## Discussion

Chemosensory transduction is under strict circadian control in insects, and previous experiments in solitary nocturnal moths suggest that the transduction cascade differs between sleep and activity states (Zhukovskaya, 1995; Linn et al., 1996; Plautz et al., 1997; Krishnan et al., 1999; Page and Koelling, 2003; Rosén et al., 2003; Flecke et al., 2006, 2010; Merlin et al., 2007; Rymer et al., 2007; Gawalek and Stengl, 2018; Dolzer et al., 2021) In *D. melanogaster* ORNs, experimental data support an ionotropic odor transduction cascade in addition to cAMP-dependent metabotropic signaling in response to general odorants (Nakagawa and Vosshall, 2009; Wicher and Miazzi, 2021). In contrast, no evidence was found for OR–Orco-dependent ionotropic pheromone transduction in the hawkmoth *M. sexta*, but evidence accumulated for G-protein–coupled activation of PLCβ (Stengl, 2010; Nolte et al., 2013, 2016; Stengl and Funk, 2013).

Although the inverse topology of insect ORs raised doubts about potential G-protein coupling (Benton et al., 2006; Wistrand et al., 2006), and some physiological studies found no evidence for G-protein–dependent cascades (Sato et al., 2008; Yao and Carlson, 2010), several physiological and neuroanatomical findings in different insect species strongly suggest that metabotropic cascades are involved in insect odor/pheromone transduction (Breer et al., 1988, 1990; Boekhoff et al., 1990, 1993; Ziegelberger et al., 1990; Stengl, 1993, 1994, 2010; Laue et al., 1997; Wegener et al., 1997; Wetzel et al., 2001; Kalidas and Smith, 2002; Kain et al., 2008; Wicher et al., 2008; Chatterjee et al., 2009; Nakagawa and Vosshall, 2009; Boto et al., 2010; Grosse-Wilde et al., 2010; Deng et al., 2011; Stengl and Funk, 2013; Ignatious Raja et al., 2014; Fleischer et al., 2018; Wicher and Miazzi, 2021). Therefore, in this study, we combined pharmacological experiments, which disrupted metabotropic signaling through different subunits of G_α_ proteins and their target, PLCβ, with tip-recordings of pheromone-sensitive trichoid sensilla in intact, healthy hawkmoths. Our qPRC results corroborate the interpretation of the electrophysiological results and provide evidence for circadian control of BAL-dependent transduction cascades.

Two lines of evidence suggested that more than one class of G-proteins is involved in pheromone transduction: the increase in the variability of the response parameters when G-protein signaling was blocked and the differences in the effects between interference with general G-protein signaling and specific PLC signaling. These findings were supported by the experiments that specifically targeted the αs and αo subunits of G-proteins, indicating that G-protein activation and cyclic nucleotides are involved in the regulation of the ORN sensitivity to pheromones.

### The highly sensitive pheromone transduction in moths is coupled to G_αo_ and PLCβ

Solitary nocturnal moth males have only a few weeks as adults to accomplish the challenging task of locating their species-specific females for mating. Thus, evolution equipped moths with an exquisitely sensitive sense of smell that apparently allows the detection of single pheromone molecules (Kaissling and Priesner, 1970; Kaissling, 1986). To do so, the system needs mechanisms for the reduction of noise in the form of other olfactory molecules and maximized odor-specific signal amplification. Patch-clamp experiments in primary cell cultures of *M. sexta* ORNs revealed the activation of three consecutive pheromone-dependent ionic currents with physiologically low concentrations of pheromone stimuli (Stengl, 1993, 1994). First, a rapid, transient, pheromone-dependent Ca^2+^ inward current activates and declines in <100 ms after the start of the pheromone response. Second, apparently based on the resulting increase in intracellular Ca^2+^, a slower Ca^2+^-gated, nonspecific cation current activates, which underlies the slower kinetics of the second component of the pheromone response. Third, a protein kinase C -dependent, slow, nonspecific cation current activates that persists for several seconds and underlies the third component of the pheromone response (Stengl, 2010). Since initially activated Ca^2+^-channel of the transduction cascade functions as a “yes-or-no trigger” for subsequent Ca^2+^-dependent amplification steps, it both obliterates noise of previous processes and amplifies the signal via Ca^2+^-dependent mechanisms.

The properties of that first Ca^2+^-channel are different from those of the OR–Orco cation channels of *B. mori* or *D. melanogaster* (Sato et al., 2008; Wicher et al., 2008), so that OR–Orco-dependent ionotropic signaling seems unlikely in *M. sexta* pheromone transduction (Nolte et al., 2013, 2016). Rather, the channels that give rise to at least the first two of the three consecutive ionic currents of the pheromone response in *M. sexta* seem to belong to the TRPC superfamily of TRP channels that are highly permeable to Ca^2+^, which were originally described in the similarly highly sensitive phototransduction cascade of *D. melanogaster* (Hardie and Minke, 1992; Gawalek and Stengl, 2018). It was discovered only recently that TRPCs are not only linked to PLCβ activation but can also be directly gated by G_i/o_ proteins (Jeon et al., 2020; Zhu, 2023). Whether this is true for the antennal TRPs of *M. sexta* remains to be examined.

In contrast to the first component, only the late, long-lasting third component of the pheromone response was linked to Orco in *M. sexta*. Therefore, a solely ionotropic mechanism of pheromone transduction, that is based on a pheromone-gated OR–Orco, is unlikely because the Orco channel does not open during the first phase of the pheromone response. It rather suggests cGMP-dependent and/or voltage-dependent Orco gating (Nolte et al., 2013, 2016).

The findings described in the current study are consistent with localization and functional analyses of G-protein subunits and PLCβ in different insect antennae. In antennae of *D. melanogaster*, RT-PCR reported the expression of six G_α_ subunits (G_s_, G_i_, G_q_, G_o_, G_f_, and concertina) in addition to different G_β_ and G_γ_ subunits (Boto et al., 2010). There, G_s_, G_i_, and G_q_ are located in the cilia of ORNs, suggesting coupling to ORs (Kain et al., 2008; Boto et al., 2010). Different physiological and behavioral assays, in combination with molecular genetics, demonstrated that G_αo_ or G_αq_ signaling is used in odor detection in *D. melanogaster* (Kalidas and Smith, 2002; Kain et al., 2008; Chatterjee et al., 2009; Ignatious Raja et al., 2014). In contrast, Yao and Carlson (2010) reported in an extensive study that G_αq_ and G_γ30A_ are involved in CO_2_ detection through gustatory receptors, but not in OR-mediated odor signaling of antennal basiconic sensilla.

Regarding moths, G_αo_, G_αq_, and G_αs_ are expressed and localized in adult antennae of *B. mori* (Miura et al., 2005). In *Antheraea pernyi*, G_αq_ was found in homogenates of the antennal ciliary fraction and, by electron-microscopic studies, was detected in cilia of pheromone-sensitive trichoid sensilla. Hence, it is likely that G_αq_ is involved in pheromone detection in *A. pernyi* by coupling to ORs (Laue et al., 1997).

Furthermore, the pheromone-dependent fast, transient rises of IP_3_ in homogenates of moth and cockroach antennae are consistent with the pheromone-dependent activation of PLCβ during the first component of the pheromone response (Breer et al., 1990; Boekhoff et al., 1993). Since pheromone-dependent rises in IP_3_ levels are decreased by interfering with G_αo_ through pTox in the antennae of insects (Boekhoff et al., 1990), it is likely that, consistent with current data in hawkmoths and our results presented here, pheromone receptors in moths use G_αo_-dependent activation of PLCβ. It remains to be tested in detail whether and how pheromone transduction of different insect ORs couple directly to G-proteins and their respective target, PLCβ, or to other enzymes such as adenylyl cyclases.

In *D. melanogaster* and other insect species, members of the cAMP signaling cascades, or receptors coupled to cAMP signaling, have been found in the antennae (Schendzielorz et al., 2012, 2015; Thamm et al., 2017; J. Li et al., 2020, Y. Li et al., 2024). They were located in ORNs, by either demonstrating the respective gene expression with molecular genetic tools or by morphological assays with immunocytochemistry. Rises in cAMP were reported after odor stimulation and mutations of the cAMP cascade results in defects in olfaction-dependent behavior, which suggests that at least some specific ORs are relying on G_αs_-dependent activation of adenylyl cyclases in *D. melanogaster* (Wicher et al., 2008; Deng et al., 2011; Wicher and Miazzi, 2021).

The discrepancies in reports of insect olfaction could stem from inconsistent experimental conditions between the different studies. Conclusions from measurements of different sensillum types and ORNs are often generalized and not distinguished in different or even the same species. Additionally, odorant concentrations and stimulus durations differ vastly between publications. Furthermore, the intracellular Ca^2+^ concentrations in the recorded ORNs are not controlled and would affect ion channel availability for odor-dependent activation (Stengl, 2010; Stengl and Funk, 2013). As we demonstrated here, interference with pheromone transduction cascades depended on ZT and mostly affected the phasic component of the pheromone response. This and previous studies indicate that the three components of the response are mediated by distinct mechanisms of the transduction cascade, and failure to address these components separately could obscure G-protein actions that seem to act mostly on the first component. Very likely, careful planning and comparative analysis of experiments across species will show that there are several redundant transduction mechanisms for insect ORs, depending on the different physiological needs of the respective insect.

In summary, our physiological data from in vivo recordings of hawkmoth trichoid sensilla are consistent with pheromone receptor-dependent activation of G_αo_, which causes activation of PLCβ4 and therefore increases of IP_3_ and diacylglycerol levels. Although BAL receptors in hawkmoth have the same inverse 7TM topology as ORs in other insects (Grosse-Wilde et al., 2006, 2007; Wicher et al., 2017), our results indicate direct or indirect coupling to G-protein–dependent amplification cascades, as shown for insect gustatory receptors and ionotropic receptors (Wetzel et al., 2001; Ishimoto et al., 2005; Nakagawa et al., 2005; Ueno et al., 2006; Stengl, 2010; Yao and Carlson, 2010). It seems that, in hawkmoths, there are several redundant or interdependent transduction cascades that guarantee the reliable, highly sensitive pheromone transduction, which depends on odor stimulus strength, duration, time course, and time of day, as well as on intracellular second messengers that vary across the day (Stengl, 2010; Stengl and Funk, 2013; Stengl and Schröder, 2021). It remains to be studied if and how receptors in ORNs and even in the non-neuronal support cells interact during pheromone and odor transduction.

### Peripheral circadian clocks orchestrate olfaction-dependent behavioral rhythms via circadian control of chemosensory responsiveness

Nocturnal male moths are maximally responsive to pheromones during their activity phase in the scotophase (Linn et al., 1996; Rosén et al., 2003; Flecke et al., 2010). These rhythms in responsiveness are controlled by circadian clocks and therefore persist under constant conditions (Baker and Cardé, 1979; Rosén et al., 2003; Merlin et al., 2007). Similarly, females, guided by their own circadian clocks, release pheromones at night and peak release correlates with the males’ search flight (Sasaki and Riddiford, 1984; Itagaki and Conner, 1988; Rosén, 2002). Mating success decreases if males and females are raised out of phase in different light/dark cycles (Silvegren et al., 2005). Both photoperiod and pheromone presence synchronize the mating behavior of moths by circadian modulation of female pheromone release and male pheromone sensitivity. How and where exactly this circadian modulation occurs in the male's olfactory system remains to be investigated further. Our results indicate that PLCβ4 is under circadian control and could be engaged in the heightened pheromone sensitivity, consistent with previous finding of circadian changes of IP_3_ levels in hawkmoth antennae (Schendzielorz et al., 2015).

Circadian clocks generate rhythms with a cycle period of ∼24 h through positive feedforward and delayed negative feedback loops. These clocks are formed at the gene transcription level as transcriptional/translational feedback loop (TTFL) oscillators or at the posttranslational level as posttranslational feedback loop (PTFL) oscillators (Stengl and Schneider, 2024). The widespread and intertwined TTFL and PTFL oscillators sustain physiological homeostasis (Stengl and Schneider, 2024) and occur not only in the central and peripheral nervous system but also in many organs throughout an organism (Plautz et al., 1997; Giebultowicz, 2000).

In response to food odors and pheromones, electroantennogram (EAG) recordings from *D. melanogaster*, the Mediterranean fruit fly *Ceratitis capitata*, and the cockroach *Rhyparobia maderae* show circadian rhythms (Krishnan et al., 1999; Page and Koelling, 2003; Merlin et al., 2007; Rymer et al., 2007; Sollai et al., 2018). Only targeted ablation of TTFL clock genes in the antenna, but not ablation of brain clocks, affected the circadian rhythms of EAGs in *D. melanogaster*, showing that the circadian oscillators in the antennae are necessary and sufficient for chemosensory rhythms (Tanoue et al., 2004). It is not well understood which mechanisms and which antennal cells control circadian rhythms in odor and pheromone sensitivity in insects, already at the periphery, and how these are synchronized to the central clock. Besides the ORNs in *M. sexta*, non-neuronal supporting cells and epithelial cells in the antennal sensilla express circadian clock genes with circadian rhythms, which suggests that many intertwined clocks and processes govern pheromone sensitivity in hawkmoths (Schuckel et al., 2007). Antennal genes that are involved in odorant binding and odorant clearance are rhythmically expressed (Claridge-Chang et al., 2001; McDonald and Rosbash, 2001; Ceriani et al., 2002; Merlin et al., 2007; Saifullah and Page, 2009; Jin et al., 2017; Dhungana et al., 2023). We could show for *M. sexta* that PLCβ4 expression depends on the time of day, which makes it a candidate for the circadian control of chemosensory sensitivity.

### Concluding remarks

The results presented here add to the emerging view that the first stage of insect olfactory transduction is not solely realized through ionotropic receptors alone but apparently involves G-protein signaling cascades. While the investigation of olfactory receptors in heterologous expression systems allows for the elimination of confounding factors and the creation of a highly controllable environment, the use of an in vivo preparation ensures that all of the naturally occurring elements of signaling pathways are present. Furthermore, temporally separating the spiking response based on the known odorant attribute that is encoded in each response component instead of pooling the whole spike train mitigates the “failure of averaging”.

## Synthesis

Reviewing Editor: Silvia Pagliardini, University of Alberta Decisions are customarily a result of the Reviewing Editor and the peer reviewers coming together and discussing their recommendations until a consensus is reached. When revisions are invited, a fact-based synthesis statement explaining their decision and outlining what is needed to prepare a revision will be listed below. The following reviewer(s) agreed to reveal their identity: Masashi Tabuchi.

The authors examined whether the olfactory signaling in olfactory receptor neurons (ORNs) of Manduca sexta sensilla involves metabotropic mechanisms alongside or instead of ionotropic mechanisms. For this purpose, they utilized the peripheral circadian clock in the chemosensory transduction process in hawkmoths. They performed in vivo electrophysiology tip-recordings of male Manduca sexta sensilla during specific times of day, which allowed them to assess the odor-evoked neural activity of ORNs. Additionally, the authors applied a more targeted disruption of Gα subunits using bacterial toxins meant to disrupt the phasic pheromone response, which helped elucidate the specific Gα subunit involved in the transduction pathway. The study also performed qPCR based on cDNA synthesis from antennae RNA extraction to allow for the annotation of the PLCβ gene in M. sexta, to determine the circadian cycling of the target gene expression.

Reviewer 1

The authors do an excellent job with presenting their methods. The figures and data are organized in a clear and concise manner, which allows for quick and easy visualization of their results. However, the study design has notable flaws that call into question the validity of the statements being made from the experimental results. Below, we are providing feedback and recommendations for revision that will strengthen the study prior to resubmission for publication.

Major Comments:

1. The authors make a comparison to D. melanogaster regarding a conserved olfactory receptor coreceptor (Orco), stating that both ionotropic and metabotropic odor/pheromone transduction cascades have been observed in the species (D. melanogaster), with no evidence of an OR-Orco-dependent ionotropic pathway found in M. sexta. Firstly, while it may be helpful to make comparison to a more well-known study specimen, it would be more beneficial to your study to make more comparisons to Bombyx mori, which is more closely related to M. sexta. This less effective comparison is made throughout the paper, particularly in the discussion, where it is more important that comparisons are made to B. mori. Secondly, it has been found that B. mori has much more detection sensitivity of sex-pheromones in their ORNs than M. sexta. However, the sex-pheromone receptor in B. mori is known to be an ionotropic (Figure 2e, https://www.nature.com/articles/nature06850 as well as Fig. 4, https://pubs.rsc.org/en/content/articlelanding/2014/cc/c3cc48216b.) Due to evolutionary relatedness, it would make sense that M. sexta would have a similar mechanism to that of B. mori, which could be stronger evidence to suggest that M. sexta does in fact utilize an ionotropic pathway.

2. Discussion section 6.2: The authors only mention ORN-based peripheral processing and leave out a discussion on central processing. In B. mori, the ability to achieve such precise detection sensitivity of the sex-pheromone is a result of contributions from central processing (https://www.pnas.org/doi/full/10.1073/pnas.1313707110), in addition to ORNs. Although we understand that ORNs have peripheral clock cycling, which may be independent from the central clock, with a large part of this study considering the circadian aspect of this process, it is important to investigate and/or discuss the central processing component, as the circadian clock is largely regulated by central processing.

3. In the discussion section, the authors make reference to multiple different studies that used different experimental assays to investigate the signal transduction system in question. Firstly, beginning from line 401, they cited the primary cell culture system from M. sexta ORNs (Stengl, 1994, 1993, 2010), which the data are not optimized to exclude the possibility of the involvement of ionotropic mechanisms. Secondly, they cited Sato et al., 2008 and Wicher et al., 2008, which are studies involving highly reliable in vitro systems. However, the authors of this paper used an in vivo system. The results of these 3 different assays (ex vivo, in vitro, and in vivo) should not be mixed together and interpreted as the same. As a result of this interpretation, the authors make claims that their data cannot strongly confirm. For example, in line 414, they state that "OR-Orco dependent ionotropic signaling seems unlikely in hawkmoth pheromone transduction." Based on the presented data from the experimental methods, they should not rule out ionotropic signaling but should suggest that ionotropic signaling alone is unlikely. Their claim of a metabotropic mechanism of pheromone transduction that is cGMP-dependent and/or voltage-dependent Orco gating cannot be strongly supported by their in vivo system. To make such claims, the authors should provide in vitro data, similar to that performed in Sato et al., 2008 and Wicher et al., 2008. If providing in vitro data is technically challenging and therefore the authors must reply on performed in vivo electrophysiology tip-recordings, they must diminish or turn down their claims. Without in vitro data, it is extremely challenging to exclude the possibility of the involvement of ionotropic mechanisms, although their in vivo data is good enough to support the involvement of metabotropic mechanisms, in addition to ionotropic mechanisms.

Minor Comments:

1. Introduction: The authors cite many papers from Kaissling, who performed studies on B. mori. They use these papers to make broad statements relating to insect/moth sex pheromone signal transduction. However, they claim that M. sexta is fundamentally different from B. mori in this pathway. In order to avoid contradiction, the authors should cite different pieces of literature.

2. Results lines 323 and 326: Adenylyl cyclase is first mentioned by name with no abbreviation as AC next to it. Line 326 uses the abbreviation AC, which is likely referring to adenylyl cyclase. Please define the abbreviation first to ensure maximum clarity.

3. Results lines 319-333: This paragraph describing the methods and reasoning behind the use of bacterial toxins to identify the Gα subunit should be incorporated into the methods section of the paper, while the Results section should focus more on the direct results of this specific experiment, which begins around line 334.

4. Discussion line 513: Change "did affect" to affected.

5. The discussion ends somewhat abruptly, stopping immediately following the discussion of the circadian control of PLCβ4 expression. It would be more effective to end with a summary paragraph that restates the findings of the entire study, particularly the results pertaining to the ionotropic/metabotropic transduction pathways. It needs to refer back to the statement of significance to prove how this study has brought about new knowledge.

Reviewer 2

L. 2-3: the sentence could be rewritten as follows: "to their respective physiological needs..." to avoid using the word "insect" twice in the same sentence

L. 42: For which odors is the other ORN specific?

L. 46: This is not true for all moths, so authors should specify and include references.

L. 49-51: This concept has already been partly expressed, so I would move it from here and reconnect it to the previous sentences.

L. 81: the authors should explain, for those who are not fully versed, what "BAL stimulation" means.

L. 86: I do not agree with the authors' assessment of phasic response. Limiting the phasic response to a precise number of spikes, rather than to the response pattern, is arbitrary. As also reported in figure 1A (AC): why not consider the entire burst of action potentials as a phasic response?

In general, I find the literature cited in the introduction to be somewhat dated. I wonder if more recent works exist. If not, might the work seem anachronistic?

L. 97: What is the composition of the diet?

L. 98: Why these breeding conditions? Usually 16:8 LD, 23{degree sign}C temperature to avoid breeding problems and higher humidity are chosen

L. 102-103: Authors should provide more information about the products purchased (purity, etc.) and suppliers

L. 112: Why was hexane chosen, which in itself already represents an olfactory stimulus?

L. 129-131: Does this mean the antenna was not whole? This did not cause short circuit problems?

L. 135-136: How could the antenna dry out if inserted into the tip of the reference electrode?

L. 146: With this application method, was the stimulation really effective?

L. 149-150: How long did you wait for them to flow to the olfactory receptor?

L. 156-157: I imagine different antennae were used

L. 205: How the authors managed to visualize spikes in DC

L. 206-207: What does this sentence mean?

L. 210: if the stimulation was 50 ms, how could the 1 s spikes be counted?

L. 212: the authors should specify what it is. Furthermore, it is also characterized by a series of mechanisms for ion transport, which could be involved in the results obtained.

L. 240-246: these are M&Ms

L. 251-257: these are not results

Why aren't the figures numbered?

L. 306-307: this is part of the discussion

5.3 paragraph: The entire paragraph should be rewritten: unless explicitly requested by the journal, all interpretations, also in light of existing literature, should be moved to the discussion section

L. 422-423: This is contradictory to what was reported above. It is not clear whether the transduction is ionotropic, metabotropic or both. The authors should explain this better.

L. 430: are we still talking about insects or mammals?

L. 510-511: similar results were also obtained in C. capitata (https://doi.org/10.1016/j.jinsphys.2018.08.007; https://doi.org/10.1371/journal.pone.0085523)

## Author Response

We cordially thank the reviewers for their constructive criticism that helped to improve our manuscript. Please see below for our responses to each item of concern.

Synthesis of Reviews:

Computational Neuroscience Model Code Accessibility Comments for Author (Required): n/a Synthesis Statement for Author (Required):

The authors examined whether the olfactory signaling in olfactory receptor neurons (ORNs) of Manduca sexta sensilla involves metabotropic mechanisms alongside or instead of ionotropic mechanisms. For this purpose, they utilized the peripheral circadian clock in the chemosensory transduction process in hawkmoths. They performed in vivo electrophysiology tip-recordings of male Manduca sexta sensilla during specific times of day, which allowed them to assess the odor-evoked neural activity of ORNs. Additionally, the authors applied a more targeted disruption of Gα subunits using bacterial toxins meant to disrupt the phasic pheromone response, which helped elucidate the specific Gα subunit involved in the transduction pathway. The study also performed qPCR based on cDNA synthesis from antennae RNA extraction to allow for the annotation of the PLCβ gene in M. sexta, to determine the circadian cycling of the target gene expression.

Reviewer 1 The authors do an excellent job with presenting their methods. The figures and data are organized in a clear and concise manner, which allows for quick and easy visualization of their results. However, the study design has notable flaws that call into question the validity of the statements being made from the experimental results. Below, we are providing feedback and recommendations for revision that will strengthen the study prior to resubmission for publication.

Major Comments:

1. The authors make a comparison to D. melanogaster regarding a conserved olfactory receptor coreceptor (Orco), stating that both ionotropic and metabotropic odor/pheromone transduction cascades have been observed in the species (D. melanogaster), with no evidence of an OR-Orco-dependent ionotropic pathway found in M. sexta. Firstly, while it may be helpful to make comparison to a more well-known study specimen, it would be more beneficial to your study to make more comparisons to Bombyx mori, which is more closely related to M. sexta. This less effective comparison is made throughout the paper, particularly in the discussion, where it is more important that comparisons are made to B. mori. Secondly, it has been found that B. mori has much more detection sensitivity of sex-pheromones in their ORNs than M. sexta. However, the sex-pheromone receptor in B. mori is known to be an ionotropic (Figure 2e, https://www.nature.com/articles/nature06850 as well as Fig. 4, https://pubs.rsc.org/en/content/articlelanding/2014/cc/c3cc48216b.) Due to evolutionary relatedness, it would make sense that M. sexta would have a similar mechanism to that of B. mori, which could be stronger evidence to suggest that M. sexta does in fact utilize an ionotropic pathway.

As requested by the referee we added more citations regarding more related species to Manduca sexta (Lepidoptera, Sphingidae) such as Bombyx mori (Lepidoptera, Bombycidae), Glyphodes pyloalis (Lepidoptera, Pyralidae), and Antherae polyphemus (Lepidoptera, Saturniidae). In addition, we added more literature on stimulus properties and sensory transduction in insects, but not on insect antennal lobe information processing, since this is not the focus of our manuscript.

We cannot confirm that sex-pheromone sensitive ORNs of M. sexta are less sensitive than ORNs of B. mori since this was not quantitatively studied so far and is not subject of this manuscript. It would require repeating the experimental studies with Tritium-labelled pheromone (E-6, Z-11 hexadecadienyl acetate HAD, Kochansky et al., 1975), combined with quantitative modeling analysis by Kasang (1971, 1973, 1974) and Kanaujia and Kaissling 1984, reviewed e.g. in Kaissling 2009 (3H-labelled bombykol, Kasang 1968; Schneider et al., 1968). Based upon patch clamp experiments on primary cell cultures of M. sexta that were stimulated with synthetic bombykal or female pheromone gland extracts (Stengl et al., 1992; Stengl 1994) it is very likely that M. sexta is as pheromone sensitive as B. mori. Cultured ORNs already started to respond when the pheromone application pipette with physiologically very low concentrations was positioned in front of the sensory neurons and very little pheromone stimulus started to diffuse into the culture medium.

There is no doubt that Sato et al., 2008, as well as others demonstrated that B. mori OR-Orco (BmOr-1 + BmOr-2) as well as OR-Orco complexes of Drosophila melanogaster, and Anopheles gambiae expressed in non-natural molecular surroundings such as different heterologous expression systems (Xenopus oocytes; HEK293T; HeLa cells) form pheromone/general odor gated unspecific cation channels via binding of the ligand to Or subunits only. These Orco homo- and heteromeres open spontaneously, even in the absence of odor/pheromone stimulation, pulling the cells´ resting potential towards their reversal potential around 0 mV.

However, it has not been proven so far that also in vivo, in their natural molecular environment in cilia of ORNs, pheromone transduction in B. mori employs an ionotropic mechanism only. So far, no other lab undertook the efforts to repeat the very thorough and systematic in vivo studies performed in M. sexta (Nolte et al., 2013, 2016; reviews: Stengl 2010; Stengl and Funk, 2013; Stengl and Schröder, 2021). Therefore, there is still no conclusive proof that also in vivo OR-Orco complexes in different species indeed all employ ionotropic mechanisms only, irrespective of their odor ligand being pheromones or general odorants. Furthermore, studies in heterologous expression systems such as Sato et al. (2008) employed very high concentrations of pheromone or odorant (10 µM;100 µM) and very long duration of stimulation in the range of seconds (10 s). These extreme pheromone stimuli are not encountered by insects in their natural environment and would lead to adaptation. However, in heterologous expression systems these strong and long stimuli were necessary to elicit any pheromone/odor responses while in the bona fide ionotropic α1 adrenergic receptor 100 nM noradrenaline was enough to elicit responses. Therefore, the pheromone and general odor stimuli employed in heterologous expression assays are very far from the natural stimuli the insects encounter in flight (Vickers et al., 2001) and it is very likely that this is due to other molecular components missing in the heterologous expression systems, but present in their respective signalosomes in vivo, such as components of metabotropic cascades.

2. Discussion section 6.2: The authors only mention ORN-based peripheral processing and leave out a discussion on central processing. In B. mori, the ability to achieve such precise detection sensitivity of the sex-pheromone is a result of contributions from central processing (https://www.pnas.org/doi/full/10.1073/pnas.1313707110), in addition to ORNs.

We did not discuss central processing of pheromone/odor detection since this is not the topic of this manuscript, which focuses on pheromone transduction in olfactory receptor enurons. However, we are aware of central processing schemes already in the antennal lobe allowing for signal to noise improvement with ~ √(2&n) , with n= number of converging ORNs. The ORNs convergence and temporal encoding allows for further increases in pheromone sensitivity in the projection neurons as shown in different insect species, which is very interesting but which is not the topic of our manuscript.

Although we understand that ORNs have peripheral clock cycling, which may be independent from the central clock, with a large part of this study considering the circadian aspect of this process, it is important to investigate and/or discuss the central processing component, as the circadian clock is largely regulated by central processing.

We agree with the referee that peripheral circadian clock neurons such as hawkmoth ORNs need to synchronize with central circadian clocks to express robust behavioral and physiological rhythms (reviewed in Stengl and Schröder, 2021). We clarified our discussion accordingly.

3. In the discussion section, the authors make reference to multiple different studies that used different experimental assays to investigate the signal transduction system in question. Firstly, beginning from line 401, they cited the primary cell culture system from M. sexta ORNs (Stengl, 1994, 1993, 2010), which the data are not optimized to exclude the possibility of the involvement of ionotropic mechanisms.

Establishing primary cell cultures of insect sensory neurons that are able to respond to pheromones at natural low concentrations and natural stimulus durations of few ms in vitro (Stengl 1986, Society for Neuroscience abstracts; Stengl and Hildebrand 1990) made it possible for the first time to examine the molecular mechanisms of odor transduction with patch clamp studies. Despite a very thorough search for directly pheromone-dependent ion channels in outside-out patch clamp recordings in none of the isolated membrane patches were pheromone responses obtained (Stengl et al., 1992). Only in the cell-attached recordings from the intact ORNs or in the first few seconds after rupture into whole-cell, before washing out cell contents, pheromone responses could be obtained (Stengl et al., 1992; Stengl 1993, 1994). Thus, already in the primary, unbiased electrophysiological studies no evidence for directly pheromone-gated ion channels were detected in moth ORNs. Instead, through a variety of different techniques employed in other labs as cited in our manuscript, there was accumulating evidence for the presence of amplifying metabotropic signal transduction cascades.

Secondly, they cited Sato et al., 2008 and Wicher et al., 2008, which are studies involving highly reliable in vitro systems. However, the authors of this paper used an in vivo system. The results of these 3 different assays (ex vivo, in vitro, and in vivo) should not be mixed together and interpreted as the same.

We agree with the referee that results from different assays should not be mixed. However, we do not agree with the referee´s opinion that in vitro systems are highly reliable and preferable over in vivo assays. We also employed different in vitro systems to examine M. sexta Orco and the hawkmoth´s bombykal receptor OR-1 (Nolte et al., 2013; Wicher et al., 2017). Our findings did not support the referee´s claim that findings from in vitro studies are much more reliable than findings from in vivo assays:

Expression of insect mRNA in heterologous in vitro systems employing vertebrate cells such as immortalized human embryonic kidney cells (HEK cells) does not necessarily allow for correctly processed insect mRNA, nor for the same post-transcriptional modification of the respective insect ion channel protein, as found in the insect in vivo. It becomes increasingly apparent that these evolutionary distant species have different processing machinery and enzymes. Furthermore, the generally employed heterologous expression systems employ membrane localization processes/molecules that are very distinct from the endogenous transport machinery of the cilium of an insect ORN. This also leads to great difficulties and unreliable outcome in the localization of expressed Orco + Ors in the HEK 293 cells plasma membranes. Our communication with other researchers confirmed that we were not the only lab with these difficulties (personal communication: Koji Sato, Jürgen Krieger, Eva Neuhaus and others...). Apparently, several other insect-specific molecules of the natural insects´ pheromone receptor complexes were missing in the various heterologous assay systems employed since even co-expression of MsexSMNP-1 did not heal the problems of membrane localization (Nolte et al., 2013). Furthermore, expression levels of Orco in heterologous expression systems needed to be carefully titrated since Orco tended to kill the cells due to Orco´s spontaneous opening and the resulting unrestrained depolarization and Ca2+ influx. From these heterologous studies we concluded that Or-Orco in vivo is constrained from opening in molecular complexes via unknown molecular components in the cilia of hawkmoth ORNs and we need to turn to in vivo assays of intact pheromone sensitive sensilla to obtain reliable answers to our research questions.

As a result of this interpretation, the authors make claims that their data cannot strongly confirm. For example, in line 414, they state that "OR-Orco dependent ionotropic signaling seems unlikely in hawkmoth pheromone transduction." Based on the presented data from the experimental methods, they should not rule out ionotropic signaling but should suggest that ionotropic signaling alone is unlikely. Their claim of a metabotropic mechanism of pheromone transduction that is cGMP-dependent and/or voltage-dependent Orco gating cannot be strongly supported by their in vivo system. To make such claims, the authors should provide in vitro data, similar to that performed in Sato et al., 2008 and Wicher et al., 2008. If providing in vitro data is technically challenging and therefore the authors must reply on performed in vivo electrophysiology tip-recordings, they must diminish or turn down their claims. Without in vitro data, it is extremely challenging to exclude the possibility of the involvement of ionotropic mechanisms, although their in vivo data is good enough to support the involvement of metabotropic mechanisms, in addition to ionotropic mechanisms.

We thank the referee for his acknowledgment of our evidence for metabotropic signaling in M. sexta. We carefully revised our wording since we agree with the referee that finding no evidence for ionotropic signaling in the hawkmoth´s pheromone responses does not exclude that, nevertheless, there could be an additional ionotropic mechanism that we did not detect.

Minor Comments:

1. Introduction: The authors cite many papers from Kaissling, who performed studies on B. mori. They use these papers to make broad statements relating to insect/moth sex pheromone signal transduction. However, they claim that M. sexta is fundamentally different from B. mori in this pathway. In order to avoid contradiction, the authors should cite different pieces of literature.

So far, it cannot be answered whether pheromone transduction in M. sexta is fundamentally different from B. mori, since different in vitro or in situ/ in vivo assays were employed for both species. We added more literature from different species as requested by the referee. Please see also our response to major comment 1.

2. Results lines 323 and 326: Adenylyl cyclase is first mentioned by name with no abbreviation as AC next to it. Line 326 uses the abbreviation AC, which is likely referring to adenylyl cyclase. Please define the abbreviation first to ensure maximum clarity.

We now consistently refer to the enzyme as adenylyl cyclase and use the acronym AC for the filtered tip recording trace to avoid confusion.

3. Results lines 319-333: This paragraph describing the methods and reasoning behind the use of bacterial toxins to identify the Gα subunit should be incorporated into the methods section of the paper, while the Results section should focus more on the direct results of this specific experiment, which begins around line 334.

Done 4. Discussion line 513: Change "did affect" to affected.

Done 5. The discussion ends somewhat abruptly, stopping immediately following the discussion of the circadian control of PLCβ4 expression. It would be more effective to end with a summary paragraph that restates the findings of the entire study, particularly the results pertaining to the ionotropic/metabotropic transduction pathways. It needs to refer back to the statement of significance to prove how this study has brought about new knowledge.

We added some brief, concluding remarks.

Reviewer 2 L. 2-3: the sentence could be rewritten as follows: "to their respective physiological needs..." to avoid using the word "insect" twice in the same sentence Done L. 42: For which odors is the other ORN specific? We expanded the sentence accordingly.

L. 46: This is not true for all moths, so authors should specify and include references. done L. 49-51: This concept has already been partly expressed, so I would move it from here and reconnect it to the previous sentences. done L. 81: the authors should explain, for those who are not fully versed, what "BAL stimulation" means.

We added a short explanation here. We expanded the BAL stimulation description in the Methods section to give more details.

L. 86: I do not agree with the authors' assessment of phasic response. Limiting the phasic response to a precise number of spikes, rather than to the response pattern, is arbitrary. As also reported in figure 1A (AC): why not consider the entire burst of action potentials as a phasic response? We do consider the whole response pattern, but we break it down into the three components (phasic response, #APs (early) und # APs (LLPR)). The first, "phasic" component lasts less than 100 ms. As we show in our results, the various blockers that we applied had significant effects on the spike frequency of the first component of the ORN pheromone response. Indeed, the phasic response does not always contain the same number of spikes in all hawkmoths tested. However, while developing automated analysis programs for the different response parameters of ORNs in Dolzer et al. (2003), we found that the limitation of the phasic response to the first <10 spikes did reveal dose response curves not significantly different from the ones with analysis of the complete duration of the phasic response. Since information content remained reliable and comparable between data sets, we settled on the first six spikes to quantify what we call "phasic response" in this manuscript.

In general, I find the literature cited in the introduction to be somewhat dated. I wonder if more recent works exist. If not, might the work seem anachronistic? For each scientific statement we attempted to give credit to the respective scientists who were the first and the best to provide the data in question. Unfortunately, often old literature is neither read nor cited. Especially in the Drosophila community, insect literature from other species is not considered and newer Drosophila studies are celebrated wrongly as first discoveries in insect neuroscience. Would you call it an anachronism if we give credit to the pioneers of scientific insights and if we step in to cross-examine whether most recent studies that dominate current views on insect odor transduction might be too simplified or even wrong due to wrong data analysis that ignores differences in information content of distinct parameters of odor/pheromone responses? We certainly do not consider our work as anachronistic since it provides new answers to apparent inconsistencies in the current literature as cited in our discussion.

L. 97: What is the composition of the diet? The diet is slightly modified from that published by Bell &Joachim, 1976, to improve animal health. We added a sentence to this section.

L. 98: Why these breeding conditions? Usually 16:8 LD, 23{degree sign}C temperature to avoid breeding problems and higher humidity are chosen The breeding conditions for our M. sexta colonies were optimized over many years by our excellent animal cultivator and result in healthy moths at high, stable numbers. Thus, we also supply other labs that are less successful to rear moths on their own (e.g., https://doi.org/10.1242/jeb.00302; https://doi.org/10.1016/j.isci.2023.106801).

L. 102-103: Authors should provide more information about the products purchased (purity, etc.) and suppliers The suppliers are listed at the beginning of section 4.2. We added information about purity to that paragraph.

L. 112: Why was hexane chosen, which in itself already represents an olfactory stimulus? Hexane is usually used for pheromone extractions from female glands. It evaporates after loading the filter paper. Please see also the dose-response curves for BAL (https://doi.org/10.1242/jeb.00302) that was stored at the same conditions as in this manuscript: Pheromone-sensitive ORNs do not respond to hexene stimulation. We added this information to the manuscript.

L. 129-131: Does this mean the antenna was not whole? This did not cause short circuit problems? Tip recording of single insect antennal sensilla is an established protocol employed successfully by several labs to record in vivo odor responses from individually identified ORNs. Details about the procedure can be read in our citations explaining how the reference electrode is inserted into the cut tip of the antenna to obtain contact with the hemolymph. If we would encounter short circuit problems, we would not be able to record any pheromone responses from single ORNs. The antennal epithelia and tissue between the recording and reference electrodes provides enough resistance to not short-circuit the two electrodes.

L. 135-136: How could the antenna dry out if inserted into the tip of the reference electrode? There seems to be a misunderstanding. As described in these very lines, and L 129 - 131, the reference electrode is inserted into the cut end of the antenna, not the other way around as this reviewer seems to have understood. Therefore, we need to seal the opening of the antenna. The cut tip of the sensillum is inserted into the glass pipette of the recording electrode and indeed does not dry out. We replaced "antenna" with "flagellum" to be more precise.

L. 146: With this application method, was the stimulation really effective? We added a reference to Dolzer et al. 2003 (https://doi.org/10.1242/jeb.00302) who demonstrated the effectiveness of this stimulation protocol with dose-response curves for bombykal.

L. 149-150: How long did you wait for them to flow to the olfactory receptor? We do not understand this question of the reviewer. As explained, we measure pheromone responses with respect to the start of the DC potential change. Thus, it is irrelevant when the stimulus arrives at the recorded sensillum. The neural response outlasts the duration of the BAL puff by several seconds (please see our explanation of the three components of BAL response in the manuscript).

L. 156-157: I imagine different antennae were used No, with a high-magnification stereo microscope it is possible under visual control to repeatedly slip the recording electrode over the same sensillum.

L. 205: How the authors managed to visualize spikes in DC We use "DC" as acronym for the unfiltered signal that has a large DC component in addition to the AC component. We added some clarification to section 4.3.

L. 206-207: What does this sentence mean? The short BAL puff elicits a spiking response that lasts several seconds. We focused on the first second of the response and evaluated the listed response parameters. We rephrased the sentence to make it clearer.

L. 210: if the stimulation was 50 ms, how could the 1 s spikes be counted? As stated above, the neural response outlasts the 50 ms BAL puff. We explained in the introduction that females release their pheromones in fast pulses. In addition, of the ubiquitous wind turbulence is further disturbing the short "scent filaments" so that the male moth never encounters a continuous pheromone stimulus in the wild.

L. 212: the authors should specify what it is. Furthermore, it is also characterized by a series of mechanisms for ion transport, which could be involved in the results obtained.

If the reviewer refers with "it" to the SPA: We define the acronym in the preceding sentence as sensillum potential amplitude, and explain in this sentence and Fig 1A how we measure it.

L. 240-246: these are M&Ms True, but we would like to start the Results with a brief summary of the methods for the readers that tend to skim the paper and skip over the Methods section.

L. 251-257: these are not results True, but this information is necessary to understand why we separate the pheromone response into the distinct components in the results section. Again, we feel that summarizing it here (we explained in more detail in the M&Ms of the manuscript) helps the reader to understand our results.

Why aren't the figures numbered? We apologize for the missing numbers. We were under the impression that they would be added by the submission software during the merging of manuscript and figures because we submitted the figures in the requested format for publication. We added low-res versions of the figures above the respective figure legends.

L. 306-307: this is part of the discussion We present this idea here because it is the rationale why we focus only on ZT 1-3 in the next section of the results.

5.3 paragraph: The entire paragraph should be rewritten: unless explicitly requested by the journal, all interpretations, also in light of existing literature, should be moved to the discussion section We have revised the paragraph accordingly and moved the interpretation of the results from the summaries to the discussion.

L. 422-423: This is contradictory to what was reported above. It is not clear whether the transduction is ionotropic, metabotropic or both. The authors should explain this better.

We have revised this statement to be clearer.

L. 430: are we still talking about insects or mammals? We are still talking about insects. The only species mentioned here is Drosophila and the literature cited here is focused on Drosophila as well.

L. 510-511: similar results were also obtained in C. capitata (https://doi.org/10.1016/j.jinsphys.2018.08.007; https://doi.org/10.1371/journal.pone.0085523) We added the citations to this paragraph.
