# Supplementary figures and images for: Hawkmoth Pheromone Transduction Involves G-Protein–Dependent Phospholipase Cβ Signaling
Source: eNeuro. 2025 Mar 5;12(3):ENEURO.0376-24.2024. doi: 10.1523/ENEURO.0376-24.2024 (PMC11964160; doi:10.1523/ENEURO.0376-24.2024)

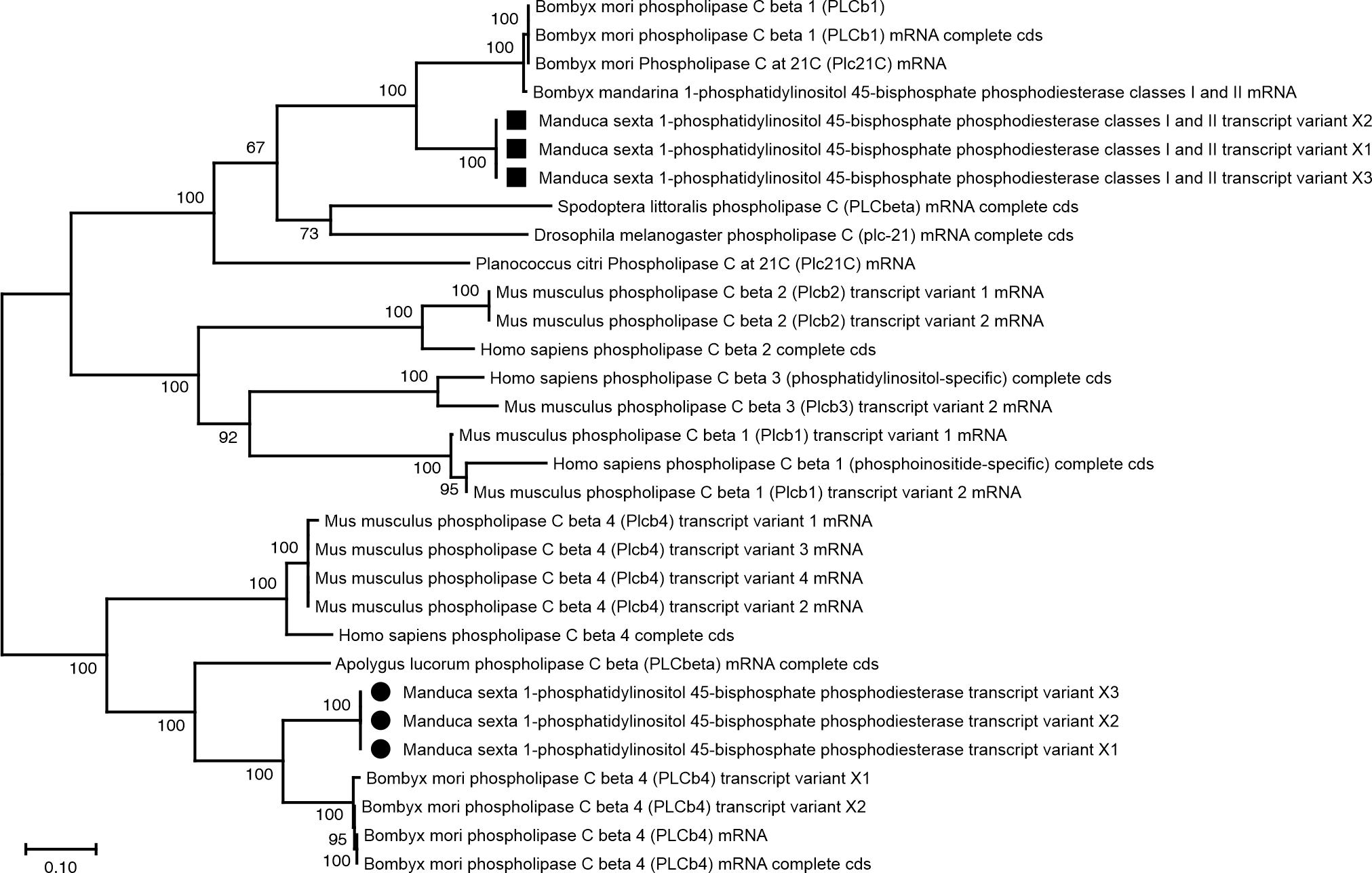

Supplement: Figure 7-1 — The phylogenetic tree was constructed using the maximum likelihood method, and node support was evaluated with 1000 bootstrap replicates. Bootstrap values > 67 are shown. Black boxes and black circles represent candidate PLCβ1 and PLCβ4 of M. sexta (Gene IDs: LOC115440592 and LOC115451385), respectively. Sequence information is detailed in Extended Data Figure 7-2. Download Figure 7-1, TIF file. [file eneuro-12-ENEURO.0376-24.2024-s005.tif]
